# Towards Extending the Detection Window of Gamma-Hydroxybutyric Acid—An Untargeted Metabolomics Study in Serum and Urine Following Controlled Administration in Healthy Men

**DOI:** 10.3390/metabo11030166

**Published:** 2021-03-12

**Authors:** Andrea E. Steuer, Justine Raeber, Fabio Simbuerger, Dario A. Dornbierer, Oliver G. Bosch, Boris B. Quednow, Erich Seifritz, Thomas Kraemer

**Affiliations:** 1 Department of Forensic Pharmacology & Toxicology, Zurich Institute of Forensic Medicine, University of Zurich, 8057 Zurich, Switzerland; Justine.raeber@bluewin.ch (J.R.); fabiosi@student.ethz.ch (F.S.); dornbierer@pharma.uzh.ch (D.A.D.); Thomas.kraemer@irm.uzh.ch (T.K.); 2 Department of Psychiatry, Psychotherapy and Psychosomatics, Psychiatric Hospital, University of Zurich, 8032 Zurich, Switzerland; oliver.bosch@bli.uzh.ch (O.G.B.); quednow@bli.uzh.ch (B.B.Q.); erich.seifritz@bli.uzh.ch (E.S.); 3 Neuroscience Center Zurich, University of Zurich and Swiss Federal Institute of Technology Zurich, 8057 Zurich, Switzerland; 4 Zurich Center for Interdisciplinary Sleep Research (ZiS), University of Zurich, 8091 Zurich, Switzerland

**Keywords:** GHB, untargeted metabolomics profiling, urine, serum, placebo-controlled

## Abstract

In forensic toxicology, gamma-hydroxybutyrate (GHB) still represents one of the most challenging drugs of abuse in terms of analytical detection and interpretation. Given its rapid elimination, the detection window of GHB in common matrices is short (maximum 12 h in urine). Additionally, the differentiation from naturally occurring endogenous GHB, is challenging. Thus, novel biomarkers to extend the detection window of GHB are urgently needed. The present study aimed at searching new potential biomarkers of GHB use by means of mass spectrometry (MS) metabolomic profiling in serum (up to 16.5 h) and urine samples (up to 8 h after intake) collected during a placebo-controlled crossover study in healthy men. MS data acquired by different analytical methods (reversed phase and hydrophilic interaction liquid chromatography; positive and negative electrospray ionization each) were filtered for significantly changed features applying univariate and mixed-effect model statistics. Complementary to a former study, conjugates of GHB with glycine, glutamate, taurine, carnitine and pentose (ribose) were identified in urine, with particularly GHB-pentose being promising for longer detection. None of the conjugates were detectable in serum. Therein, mainly energy metabolic substrates were identified, which may be useful for more detailed interpretation of underlying pathways but are too unspecific as biomarkers.

## 1. Introduction

In clinical and forensic toxicology, gamma-hydroxybutyrate (GHB) still represents one of the most challenging drugs of abuse (DOA) in terms of analytical detection and interpretation. Next to its recreational consumption as a party drug, its sedative properties combined with its chemical characteristics (i.e., colorless and almost odorless; easy to mask slightly soapy taste) makes it particularly suitable to be misused as a so-called *date rape* or *knock-out* drug in cases of drug facilitated crimes (DFC) or drug facilitated sexual assaults (DFSA). The fact, that GHB can cause short-term anterograde amnesia also helps the perpetrator [1,2]. Further to its illegal use, GHB is approved for the treatment of narcolepsy with cataplexy, given its ability to consolidate sleep, reduce excessive daytime sleepiness and the prevalence of cataplectic attacks [3]. As a short-chain fatty acid, GHB is also an endogenous compound, produced through the degradation of the inhibitory neurotransmitter gamma-aminobutyric acid (GABA) and a precursor of succinic semialdehyde (SSA). SSA is subsequently oxidized to succinate, an intermediate of the Krebs cycle [4,5].

GHB is structurally different to common DOAs such as the basic drugs cocaine, heroin, and amphetamines and is usually not covered in routine screening procedures. This means, that additional methods such as gas chromatography-mass spectrometry (GC-MS) after derivatization or liquid chromatography-MS (LC-MS) in negative ionization modes are required for its detection [6]. Due to its rapid elimination, the detection window of GHB in common matrices is short, with maximum 6 h in plasma and 12 h in urine, respectively [7,8]. Mainly in DFSA cases, the detection of GHB intake, particularly the differentiation of low exogenous GHB remaining from a previous intake from naturally occurring endogenous levels, is challenging [1]. In contrast to other classic DOAs, where detection of drug metabolites usually extents their detectability, GHB lacks such unambiguous metabolites.

Thus, a major focus in last years has been on phase II metabolites of GHB, such as sulfate and glucuronide conjugates, which were thought to present longer detection windows [9,10]. Anyhow, to date reliable methods discriminating whether GHB-glucuronide or GHB-sulfate are derived from exogenous or endogenous sources are lacking [9,10,11,12]. Recently, research interest moved to targeted and untargeted evaluation of endogenous metabolites of GHB resulting either from GHB beta-oxidation or its initial formation to SSA [13,14,15,16]. Based on former findings of elevated levels 2,4- and 3,4-dihydroxy butyric acid [17,18], Jarsiah et al. recently developed a method to quantify these metabolites together with other organic dicarboxylic acids glycolate and succinate in blood and urine samples of GHB users. However, the actual influence of GHB on the organic acids in real consumer samples has not yet been published [15]. Luca et al. reported up to sevenfold higher levels of glycolate in mice brain and liver samples [16]. Similar results were described by Palomino-Schätzlein et al., who showed significant increases in glycolate and succinate levels in human urine samples collected in a controlled GHB administration setting [14]. Using untargeted metabolomics profiling in a former study, we were also able to show increased levels of glycolate and succinylcarnitine, and additionally identified new conjugates of GHB with carnitine and the amino acids glutamate and glycine [13]. However, strict filter criteria were applied on urine samples, thus not fully exploiting the possibilities of the data set. In addition, only urine samples collected as early as 4.5 h after GHB intake were analyzed. Nevertheless, this and the aforementioned studies highlight the general advantages of targeted and untargeted metabolomics techniques for identification of endogenous metabolites of GHB.

Particularly untargeted metabolomic strategies generally aim for high-throughput identification of a multitude of small molecular weight molecules (<1000 Da) affected by a certain intervention, e.g., drug consumption. In terms of DOAs including GHB, this has been mainly applied to gain deeper insights into pharmacological properties of a drug, discover new metabolites in a time- and dose-dependent manner or identify and interpret pathways implicated in the mechanism of drug action, adverse effects and variability of the drug response [12,14,16,19,20,21].

Despite recent studies with promising findings, new biomarkers for improved and extended detection and interpretation of GHB consumption are urgently needed. To start filling this gap, the aim of the present study was to search for and evaluate potential new metabolites or endogenous biomarkers of GHB consumption, by means of a metabolomic profiling approach. We significantly extended our previous work by finally including for the first time both serum (collected before, 4.5 h and 16.5 h after intake) and urine samples (collected 4.5 h and 8 h after intake) collected in the framework of two randomized, placebo-controlled, crossover study investigating the neurobiological effects of GHB in 15 and 20 healthy male volunteers, respectively. While the previous work represents a simple proof-of-concept study, with significant limitations such as short-term urine samples (only up to 4.5 h) and the lack of blood samples for analysis, the current studies clearly focus on the forensic relevant question of time-windows of possible new biomarkers highlighting and, for the first time, comparing results from urine samples collected later than 4.5 h and finally bloodserum samples up to 16.5 h post GHB intake.

## 2. Results

### 2.1. Analytical and Data Processing Procedures of Samples from a Controlled Clinical Study

Based on our previous experience [13,22,23], a state-of-the-art untargeted metabolomics approach was used applying LC-quadrupole time of flight (qTOF)-MS analysis in four different operating modes (reversed phase chromatography [RP] and hydrophilic interaction liquid chromatography [HILIC] in combination with electrospray ionization (ESI) in positive and negative polarity mode, respectively) to obtain abundances (counts per seconds, cps) of a multitude of endo- and exogenous metabolites [24]. MS/MS experiments were performed on selected samples in addition to full scan analysis for identification purposes. Serum and urine samples collected after randomized, placebo-controlled, crossover administration of GHB [25] were analyzed in randomized order and analytical performance controlled by regular system suitability tests (SST) and pooled plasma quality control samples (QC). Mean coefficients of variation (%CV) values of all compounds in the SST and median CV of all features exceeding 500 cps in the QC were within our acceptance criteria of <30% in all four methods.

Serum samples from 20 male volunteers were collected prior to the intake of the study medication at night in the middle of a sleep episode (t0) and then 4 h (t1) and 16.5 h (t2) after. Urine was sampled in the morning, approximately 4.5 h after GHB or placebo intake (Ut1) [25]. Additionally, early morning urine samples from an initial GHB pilot study from 15 male volunteers, where the study medication was administered in the evening prior to sleep, were collected in the morning, approximately 8 h after dosing (Ut2).

For data evaluation, Progenesis Qi software was applied on MS data sets for retention time (RT) alignment, peak picking and data deconvolution (grouping of adducts of the same compounds). A list of all detectable features (defined by their RT and accurate mass) was generated and submitted to further statistical evaluation to identify promising biomarkers formed or significantly changed by GHB intake as described in detail below. Identification was performed by common database searches, applying the *Fragment similarity search* tool from Metlin (https://metlin.scripps.edu (accessed on 24 November 2020)) or by a priori MS interpretation.

### 2.2. (Un)targeted Metabolomics Profiling of Urine Samples 4.5 h and 8 h Post GHB Administration

**Figure 1:**
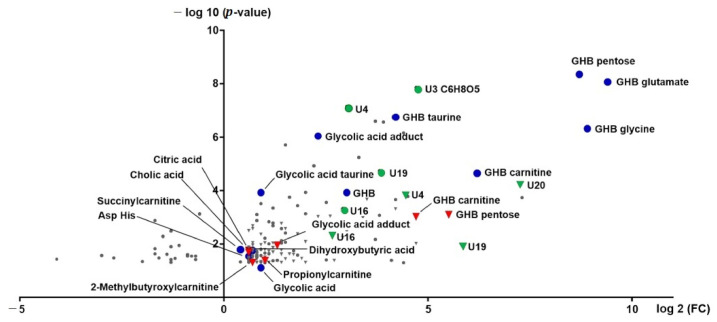
Volcano plot of urinary features 4.5 h (circles) and 8 h (triangles) after administration of GHB. Identified features are indicated in blue (4.5 h) and red (8 h), potentially interesting, but as of yet unknown features are given in green.

Our initial biomarker search performed on urine samples collected approximately 4.5 h post GHB intake and evaluated using string filtering criteria instead of exploiting the whole dataset identified three potential biomarker for GHB intake: GHB-carnitine, GHB-glycine and GHB-glutamate [13]. In the current study, the urinary data after 4.5 h in addition to urine samples collected approximately 8 h post GHB intake were submitted to global metabolomics profiling. All features with peak intensities exceeding 500 cps were considered after normalization to 100 mg/dL creatinine and volcano plot analysis (paired t-test, *p* < 0.05, fold-change (fc) > 1.5 in at least 70% of sample pairs). Features with similar retention times (± 0.3 min) in the same chromatographic mode (RP or HILIC) and/or similar accurate masses (± 20 ppm) of full scan precursor ions or fragment ions were manually evaluated in all methods for both time points and combined to feature groups, respectively. In total, 156 features/feature groups were selected and are depicted as volcano plot (log 2 fc vs. −log 10 *p*-value) in Figure 1. An overview of all features and manually build feature groups numbered and sorted in order of decreasing significance is given in the supplementary information Table S1. From all features/feature groups, 17 could be at least tentatively identified. Mass spectra of selected, tentatively identified biomarkers together with the proposed chemical structure and fragment interpretation are depicted in the supplementary information Figure S1. In addition to the metabolites already described in the previous study (GHB-carnitine, GHB-glycine, GHB-glutamate, glycolic acid) more conjugates could be identified as GHB-pentose and GHB-taurine. GHB-pentose and GHB-glutamate thereby provided the highest significance combined with the greatest fc. Further conjugates of glycolic acid—an unknown glycolic acid adduct (U11, *m*/*z* 159.0106) and glycolic acid taurine—also significantly increased in the GHB group compared to placebo. The respective mass spectra of glycolic acid, the unknown adduct, and glycolic acid taurine can be found in Figure S1. For definite identification purposes, glycolic acid was compared to a reference standard measured under the same conditions.

Next to the expected *m*/*z* of 75.0084 for glycolic acid, the full scan MS and MS/MS indicated an additional *m*/*z* in RP neg analysis of *m*/*z* 159.0106, matching in accurate mass and MS/MS spectrum to the unknown feature U11 and displaying the same chromatographic behavior as glycolic acid. The exact structure of this glycolic acid adduct, however, remained unknown. All other compounds identified, namely dihydroxybutyric acid, citric acid, cholic acid, succinylcarnitine, and propionylcarnitine fulfilled the criteria while the overall difference between placebo and GHB was much lower than for the aforementioned compounds. Overall, a large amount of promising features/feature groups remained unidentified. For instance, feature U3 with large fcs, and the features/feature groups U4, U16, U19, and U132 appear interesting, the later ones showing similar MS/MS spectra with masses *m*/*z* 72, 100, 169 as depicted in Figure S2.

In addition to the untargeted search strategy, targeted search was applied screening the selected feature list (Table S1) for the following compounds: conjugates of GHB with all proteinogenic amino acids, of GHB and its oxidation products SSA, succinic acid, GABA, glutamate, dihydroxybutyric acid, and glycolic acid with carnitine, taurine, glycine, glutamine and glutamate. However, no features matching to any of the aforementioned possible GHB conjugates could be detected.

**Figure 2:**
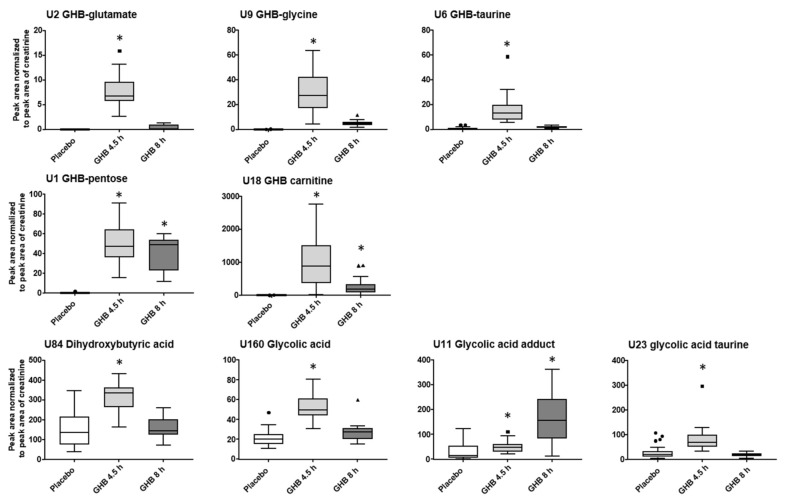
Blox plots (outliers given as circles, squares or triangles for placebo, 4.5 h and 8 h post GHB intake, respectively) for selected, identified compounds representing typically observed changes between placebo and two time-points after GHB intake. Depicted are analyte peak area to creatinine peak area ratios for placebo (*n* = 34) and GHB groups after 4.5 h (Ut1, *n* = 19) and 8 h (Ut2, *n* = 15). Detection using univariate volcano plot analysis (*p* < 0.05 and fc > 1.5 in at least 70% of the samples) is indicated by an asterisk.

**Figure 3:**
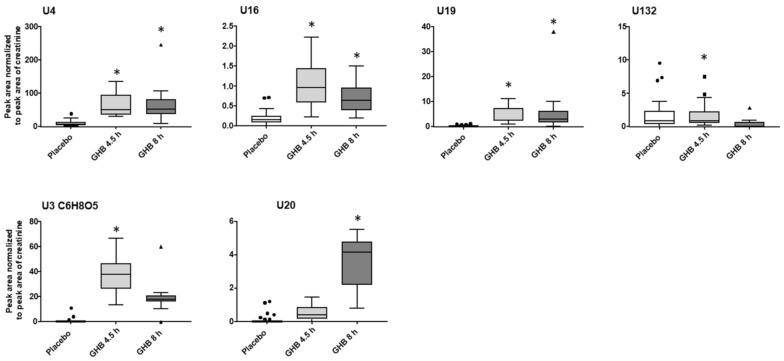
Blox plots (outliers given as circles, squares or triangles for placebo, 4.5 h and 8 h post GHB intake, respectively) for selected promising, but still unidentified, compounds representing typically observed changes between placebo and two time-points after GHB intake. Depicted are analyte peak area to creatinine peak area ratios for placebo (*n* = 34) and GHB groups after 4.5 h (Ut1, *n* = 19) and 8 h (Ut2, *n* = 15). Detection using univariate volcano plot analysis (*p* < 0.05 and fc > 1.5 in at least 70% of the samples) is indicated by an asterisk.

The initial urinary metabolomics profiling was done independently of the time interval between GHB intake and urine collection. From the filtered data set, 13 features significantly changed with fc > 1.5 after 4.5 h and 8 h post GHB dose, among them GHB-carnitine, GHB-pentose, U4, U16 and U19 (Figure 1, Table S1). Additionally, all identified and potentially interesting features/feature groups at either 4.5 h or 8 h were manually searched for at the respective other time point. Peak areas normalized to the respective peak area of creatinine for GHB amino acid conjugates, GHB-pentose, GHB-carnitine and the organic acids dihydroxybutyric acid, glycolic acid, and the glycolic acids adduct and taurine-conjugate and are given in Figure 2. Only GHB-conjugates and the unknown feature U3 (Figure 3), but not dihydroxybutyric acid nor glycolic acid and its analogues, were largely undetected under placebo conditions.

Except for the yet unknown glycolic acid adduct, all urinary compound levels decreased from 4.5 h to 8 h samples, whereas from all conjugates, GHB-pentose tended to decline the fewest. Most features/feature groups, including e.g., amino acid conjugates of GHB, would have remained undetected 8 h post GHB intake within the untargeted workflow. Manual search however still confirmed their presence in urine samples. The compounds U4, U16, U19 showed relatively stable area ratios after 4.5 h and 8 h compared to the low amounts in the placebo group as depicted in Figure 3. Feature U132, with similar MS/MS fragmentation as U4 and U16 (Figure S2), in contrast, revealed overall low discrimination power between placebo and Ut1 and Ut2 post GHB intake. Compound U20 apparently marks the only feature with higher urinary levels 8 h post GHB intake compared to the 4.5 h samples, indicating its potential as a possible long-term marker. GHB itself was detected in Ut1 and Ut2 (with one exception) samples, with lower levels after 8 h, and only traces in the placebo samples (Figure S3).

### 2.3. (Un)targeted Metabolomics Profiling of Serum Samples

**Figure 4:**
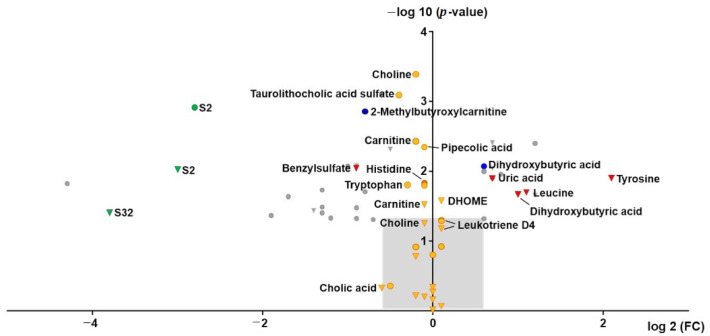
Volcano plot of features in serum 4.5 h (circles) and 16.5 h (triangles) after administration of GHB. Identified features are indicated in blue (4.5 h) and red (16.5 h), potentially interesting, but as yet unknown features are given in green. Features additionally detected by application of mixed-effect model statistical calculation are depicted in orange. Further unknown features are given in grey. The light grey area indicates the range outside the volcano plot selection criteria (−log10 *p*-value < 1.3; log2 fc < 0.6 or > −0.6).

The same filter and grouping criteria as for urine samples were applied as described above separately for t1 (4.5 h post GHB) and t2 (16.5 h post GHB), respectively. An overview of the selected features/feature groups is given in Table S2 of the supplementary information. In total 31 features/feature groups were selected independently of time and are depicted as volcano plot (log 2 fc vs. –log 10 *p*-value) in Figure 4. Only few substances could be identified including dihydroxybutyric acid, 2-methylbutyroxylcarnitine, uric acid, tyrosine and leucine. Largest fc could be observed for features S2 and S32, but both remained unidentified. Mass spectra of S2 and S32 are given in Figure S4 of the supplementary information. However, when also comparing peak areas prior to dosing (t0), the same effect was observed independent from GHB administration. 

Therefore, in addition to classic univariate analysis a mixed-effect model with increased statistical power, based on baseline correction and log-transformation, was applied. An overview of features additionally detected and identified according to the MSI as confidence level 1 or 2 is given in Table S3. In total, 27 compounds were additionally identified, mainly belonging to the compound classes of amino acids, carnitines, phosphatidylcholines, bile acids and fatty acids. Overall, without baseline correction and log-transformation these compounds show only little differences (fc) between placebo and GHB sessions as indicated as orange circles (t1) and triangles (t2) in the volcano plot (Figure 4). For example, choline was highly significantly affected by GHB intake both after 4.5 h and 16.5 h, while median fc was only 0.9.

Compared to the urinary metabolomics profiling, only dihydroxybutyric acid and 2-methylbutyroxylcarnitine appeared also affected by GHB intake in serum after univariate statistical analysis. To avoid missing compounds in serum due to expected lower concentrations, additionally, hits from urinary analysis, were searched manually in serum samples. However, except for traces of GHB-glycine, none of the aforementioned urinary biomarkers could be detected.

**Figure 5:**
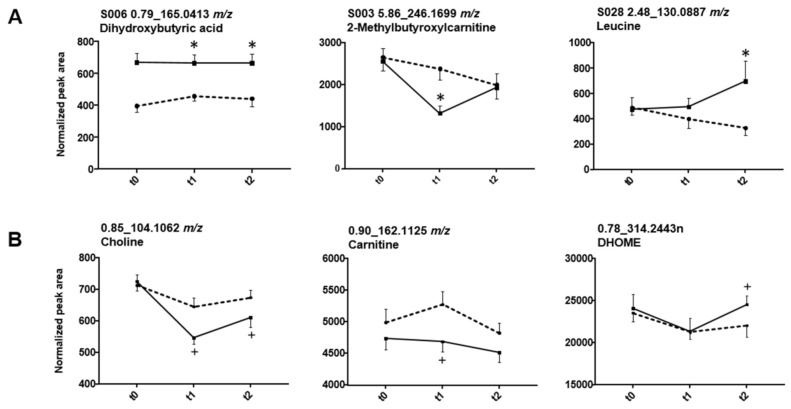
Influence of GHB on selected compounds detected using univariate statistics (volcano plot) (**A**) and mixed-effect model statistics (**B**). Solid lines indicate drug, broken lines placebo intake, respectively. Data points represent mean and standard error of mean (SEM) of 19 replicates. Detection using univariate volcano plot analysis (*p* < 0.05 and fc > 1.5 in at least 70% of the samples, (**A**) is indicated by an asterisk. Significant changes (*p* < 0.05) observed by mixed-effect model calculations (**B**) are highlighted with +.

Again, initial biomarker search was performed independent of the time passed since administration. While univariate analysis indicated 21 features/feature groups to be significantly changed at timepoint 1, 12 were found to be affected after 16.5 h (t2), and only three at both 4.5 h and 16.5 h (dihydroxybutyric acid, S2, and S4). As depicted in Figure 5A, e.g., 2-methylbutyroxylcarnitine decreased 4.5 h post GHB dose but returned to similar levels as in the placebo session till 16.5 h. On the contrary, leucine showed no significant difference between placebo and GHB at t1, but after 16.5 h. Tyrosine behaved similarly to leucine, with significant differences only after 16.5 h. The additional amino acids detected only using the mixed-effect model (Table S3) were only slightly different after 4.5 h but no longer after 16.5 h. From all compounds found based on mixed-effect model calculation, time-courses are exemplarily depicted for choline, carnitine and 9,10-DHOME in Figure 5B.

In total, 15 compounds were significantly different after 4.5 h (therefrom 3 amino acids and carnitine) and only 2 after 16.5 h (9,10-DHOME and carnitine). Only for carnitine, significant changes could be observed at both time-points. The remaining 11 compounds showed only significant differences after baseline correction and were excluded for further interpretation. GHB itself was detected neither by univariate nor by mixed-effect model statistics. Results from manual peak integration are given in Figure S3, indicating increased levels at 4.5 h in the GHB session.

## 3. Discussion

Despite constantly ongoing research efforts, the reliable detection and forensic interpretation of GHB consumption remains an unsolved problem. Still, new (bio)markers are necessary, to extent the window of detection and to improve the differentiation between low concentrations of exogenous from endogenous GHB. (Un)targeted metabolomics is currently extensively discussed as a promising new technique also for forensic toxicological applications and biomarker search of DOA consumption [20,26]. While recent studies in serum or blood samples were able to identify metabolic changes caused by GHB intake, the observed difference were subliminal and insufficient to proof drug intake [20,23,27]. Urinary analysis following GHB administration however revealed previously unknown GHB-conjugates with carnitine, glycine and glutamate and confirmed these in authentic samples [13]. This proof-of-concept study suffered from limitations such as use of urine samples collected only up to 4.5 h after GHB intake and blood samples were completely missing. While these limitations still allowed the basic proof-of-concept, a new study was needed to tackle real world forensic problems with GHB being used for DFCs. Consequently, urine samples collected at a later time-point (8 h) following GHB intake were used and, for the first time, serum samples for up to 16.5 h post GHB consumption were included and evaluated. This sampling regimen is far more representative for authentic cases, where samples are often not available in the first few hours after GHB application.

Although urine represents the matrix of choice for screening purposes [28,29] and so far, appeared superior in metabolomic profiling of DOA influences, in many laboratories, (quantitative) toxicological analysis relies on blood samples only. While in general, later time-point samples are beneficial to consider the possibility to extent the detection window earlier samples might be superior for initial biomarker search. Thus, the combination of blood and urine collected at two time-points each should enlarge the opportunity for new biomarker identification. Nevertheless, it needs to be noted, that the controlled clinical studies were not initially planned for the herein presented metabolic profiling, but for other psychopharmacological research questions. Therefore, particularly urine sampling beyond the time windows covered herein was unfortunately not possible. For future clinical studies, corresponding amendments are planned.

Next to univariate statistics (volcano plot analysis), mixed-effect model statistics were performed assuming a random effect for the crossover design of the study. While the mixed-effect model increased the statistical power and allowed the detection of more features in blood affected by GHB consumption compared to univariate statistics, the findings indicated only little differences between placebo and GHB session (Figure 4). This makes the identified features interesting for further in-depth investigation of the underlying mechanisms of GHB action, but less suitable as biomarkers for improved GHB detection, as for authentic samples, baseline samples prior to drug intake/administration are usually unavailable.

Overall, markers from different groups or subgroups (GHB conjugates, organic acid and their secondary metabolites, amino acids, carnitines, bile and fatty acids, etc.) were identified in blood or urine and will be discussed in detail in the following. GHB-sulfate and GHB-glucuronide were already shown as unreliable to extent the detection window in recent studies [11,12,13] and were, therefore, not further evaluated herein.

### 3.1. GHB Amino Acid Conjugates

Already in our previous proof-of-concept study, two conjugates of GHB with the amino acids glycine and glutamate were described [13]. Now, additionally GHB-taurine (U6, 4.63_210.0442 *m*/*z*) was identified. At the same RT, feature 4.67_539.2039 *m*/*z* could be detected, that fragments to *m*/*z* 210.04250 (C_6_H_12_NO_5_S), the precursor mass of GHB-taurine. However, it remains unclear what triggered the neutral loss of *m*/*z* 329 and whether it is an analytical artifact/adduct of GHB-taurine or an endogenous metabolite. In general, conjugation of carboxylic acids with amino acids is known as an important metabolic biotransformation by forming a peptide or amide bond between the amino group of the amino acid and the carboxylic moiety of the xenobiotic acid [30]. Amino acid conjugation has been shown for various different amino acids, whereas the chemical structure of the carboxylic acid is responsible for the definite amino acid conjugate. The most frequently observed amino acids conjugated in humans are glycine, glutamine and taurine [30,31,32]. Glutamine conjugates, as well as all other amino acid conjugates were not detected for GHB even after targeted search of expected M-H precursor ions. GHB-glutamate and GHB-glycine were not detected in samples from the placebo session while traces of GHB-taurine could be identified. All three conjugates markedly increased in urine samples 4.5 h post consumption, but rapidly declined in the 8 h urine samples (Figure 2). Considering the relatively low abundance, it seems questionable whether the analysis of amino acid conjugates can actually extent the detection window in urine. In serum samples, only traces of GHB-glycine could be detected in random samples, but did not allow for integration and comparison between serum collection time-points. More sensitive, quantitative methods and respective reference standards will be necessary to search for amino acid conjugates in blood samples.

### 3.2. Other GHB-Conjugates, GHB-Carnitine and GHB-Pentose

GHB-carnitine, the conjugate of GHB with l-carnitine, has already been detected in urine of the initial study 4.5 h post GHB intake [13]. The feature group U1 with highest significance and fc (Figure 1, Table S1), showed a classic neutral loss of *m*/*z* 132 to *m*/*z* 103.0395 suggestive of a pentose (e.g., ribose) conjugate of GHB (Figure S1). The enzyme responsible for ribose conjugation is adenosine diphosphate (ADP)-ribosyltransferase, which is proposed to transfer ADP from e.g., nicotinamide adenine dinucleotide (NAD) or nicotinamide adenine dinucleotide phosphate (NADP) to the xenobiotic substrate followed by phosphatase hydrolysis [33]. Similar to glucuronidation or glycosylation, ribose conjugation leads to the formation of more polar metabolites. Ribose conjugation represents a rather uncommon metabolomic reaction, but had been observed e.g., for rifampicin [33] or diclofenac [34]. So far, it was mainly observed in rodent species, but not in higher species such as humans [33]. While GHB-carnitine, similarly to GHB amino acid conjugates rapidly decreases in abundance already in urine samples 8 h after consumption, levels of GHB-pentose appeared only slightly lower 8 h after intake compared to the earlier sampling. This makes GHB-pentose the most promising marker among the so far detected GHB conjugates to extent the detection window of GHB.

### 3.3. Unknowns Features/Feature Groups U3, U4, U16, U19, U20

The ideal biomarker should be undetectable in placebo samples and highly abundant following GHB administration. Next to the aforementioned GHB-conjugates, this also applied for some, yet unknown, urinary compounds (Figure 3), for instance for U3. Its chemical composition was postulated as C_6_H_8_O_5_ based on its accurate mass; commercial database search suggested an MS/MS match to oxoadipic acid, which could however not be confirmed by the analysis of the reference standard. In addition, U4, U16, and U19 appeared quite promising, particularly for their low decline from 4.5 h to 8 h after GHB intake (Figure 3, upper panel). Based on their similar MS/MS information (Figure S2) these feature groups should contain similar functional groups or common substructures. Application of the MS/MS similarity search in Metlin pointed to either tripeptide (sub)structures or purine analogues, but the real chemical structure explaining all three fragments ions of *m*/*z* 72 (C_3_H_6_NO), *m*/*z* 100 (C_5_H_10_NO) and *m*/*z* 169 (C_9_H_17_N_2_O) could not be elucidated. The presence of low amounts also in placebo samples and the feature U132, which apparently showed only little differences between placebo and GHB, contradicts (in contrast to U3) the assumption of direct GHB derivatives or conjugates. Finally, U20 was one of few compounds that increased significantly after 8 h, but not after 4.5 h post intake. However, none of these compounds will be suitable as discriminants for GHB intake as long as the exact chemical structures remain unknown. Without unambiguous identification it cannot be excluded, that newly formed features originate from other ingredients of the administered Xyrem^®^ preparation, such as e.g., the adjuvant malic acid.

### 3.4. Organic Acids (Glycolate, Succinylcarnitine, Dihydroxybutyrate)

While the aforementioned conjugates of GHB were largely undetectable in the placebo session samples, organic acids or follow-up metabolites of those, could be detected in both sessions, but with significant differences. Organic acids have already been discussed by different authors as elevated after GHB intake [13,14,15,16,17,18]. Succinylcarnitine and glycolate were already determined in the initial biomarker search investigation [13]. Additionally, the conjugate of glycolic acid with the amino acid taurine was newly identified in urine 4.5 h post GHB consumption, which higher significance but similar fc compared to glycolic acid (Figure 1). However, the lack of a commercial reference standard makes it less favorable for routine investigations compared to glycolic acid itself. Feature U11 was also considered to be a derivative or adduct of glycolic acid. The exact chemical composition of this feature remains unknown; however, it was not only detected by volcano plot analysis in urine samples post GHB intake, but also when analyzing the reference standard of glycolic acid. Being only present in RP (solvent methanol, acidifier formic acid), but not in HILIC mode (solvent acetonitrile, acidifier acetic acid) might lead to the conclusion, that it is a matter of a formic acid or methanol adduct. Different structural possibilities were calculated, with the formic acid adduct of glycolic acid dimerization and cyclization under elimination of two water molecules being the closest to the observed nominal mass to charge ratio (Figure S1). However, the accurate mass information did not match the postulated structure. Interestingly, both glycolic acid and glycolic acid taurine behave alike in urine samples with increased concentration after 4.5 h but levels in similar ranges to placebo conditions after 8 h. The unknown adduct of glycolic acid showed an opposite effect, with higher level in the 8 h samples (Figure 2). Still, without final structural characterization, it cannot be unambiguously confirmed, that it is actually a derivative of glycolic acid and whether it might be useful as such to extent the detection window of GHB. As already discussed in former studies, using glycolic acid alone most likely lacks specificity as a proof for GHB intake given its presence in all samples and possible other sources for increased concentration, such as e.g., ethylene glycol poisoning [13,15]. In our data set, dihydroxybutyric acid, the oxidation product formed through alpha- and beta-oxidation of GHB was indicated as statistically significant after GHB intake. Herein, dihydroxybutyric acid was one of the few compounds, which was found not only in urine, but also in serum samples (Figure 1 and Figure 4). In urine analysis (Figure 2), time-dependent observations were similar to other organic acids like glycolic acid or succinylcarnitine indicating an increase caused by GHB intake. Unfortunately, in serum samples (Figure 5), also higher levels were found in t0 samples, collected prior to placebo or GHB intake, which points to an effect independent of GHB administration. The current analytical method was not optimized for chromatographic separation of GHB or its metabolites and was unable to separate the two isomers 2,4- and 3,4-dihydroxybutyric acid. Previous studies though indicated different concentrations of both isomers in humans, with higher concentrations of the 3,4-isomer in blood and urine [15].

### 3.5. Endogenous Changes of Amino Acids, Carnitines, Tricarboxylic Acid Cycle Intermediates, Bile Acids, Fatty Acids

Particularly in the studied serum samples, a number of amino acids, acyl-carnitines, intermediates of the tricarboxylic acid cycle (TCA), bile and fatty acids have been identified as affected by GHB. The majority of these endogenous compounds relates to energy metabolism in the broadest sense and have also been described in relation to consumption of other DOAs [20,23,35,36,37,38]. As such, their suitability as biomarkers for identification of GHB in particular seems questionable, as first the observed fcs were small, particularly considering the highly controlled conditions of the study, and second they seem unlikely to allow differentiation of GHB intake from other DOAs. While for general screening of DOA consumption this may be sufficient to trigger further confirmatory analysis, for definitive proof of GHB administration it is unfeasible.

Our study had some limitations. The study design did not mimic typical DFSA case circumstances: only men were allowed as study participants, while victims of DFSA cases typically are women (with a few exceptions); GHB was administered in form of the pharmaceutical Xyrem^®^. In authentic cases of drug users, often gamma-butyrolactone is used as basic material (bought as cleaning agent) and sometimes it is hydrolyzed to GHB before use. The used dose in the study represented the maximal starting dose used for the treatment of narcolepsy (compendium.ch), but still was likely moderate to low compared to doses administered in DFSA cases. Although urine samples were collected at later time-points (8 h) compared to the initial proof-of-concept study, they were still within the detection range of GHB itself (Figure S3). The results from urine samples are therefore not yet sufficient to proof an elongated detection window. Results from serum samples would have been suitable for such conclusions, but unfortunately, no promising marker was identified in the 16.5 h serum sample. Differences between placebo and GHB observed at that time-point, e.g., leucine, tyrosine or carnitine were too low for routine discrimination. Overall, urine still was the superior matrix for biomarker search purposes and revealed several promising new biomarkers. These biomarkers now need to be synthesized as reference material and incorporated into validated, quantitative methods for further evaluation of their behavior over longer time ranges and in authentic samples and to characterize their short- and long-term stability, sensitivity and selectivity under routine conditions.

## 4. Materials and Methods

### 4.1. Study Design and Sample Collection

The study used a randomized, balanced, double-blinded placebo-controlled crossover design given in detail in reference [25]. It was approved by the Cantonal Ethics Committee of Zurich (BASEC nr. 2016-00184) and the Swissmedic, was registered at ClinicalTrials.gov (NCT02342366) and performed according to the Declaration of Helsinki. All participants were informed about potential risks concerning of GHB, provided written informed consent and were paid for their participation in the study.

Between two sessions (placebo and GHB) a washout phase of seven days was maintained. Each session included three nights: an adaptation night, followed by the experimental night—where patients were administered either placebo or 50 mg/kg bodyweight of sodium oxybate (Xyrem^®^)—and, finally, a follow-up night. None of the participants (*n* = 20, male, healthy, mean age 25.8 ± 2.45 years reported previous experiences with GHB in their life. All were non-smokers and had to restrain from illegal drugs for two weeks (controlled by a negative drug test), from caffeine one week prior to the first until the second experimental night and alcohol was not allowed 24 h before the experimental nights.

In the main study, administration took place at 2:30 a.m. of the experimental night. Subjects were woken up and each subject received placebo and 50 mg/kg of GHB (Xyrem^®^) dissolved in 2 dL of orange juice (each in two different session). Both matched in appearance and taste. Blood samples were collected in serum separating tubes containing clot activator and separation gel (BD Vacutainer^®^, Becton Dickinson AG) prior to the experimental night at 7:00 p.m. (t0, −7.5 h) and after GHB/placebo administration at 7:00 a.m. (t1, 4.5 h) and 7:00 p.m. (t2, 16.5 h) after the experimental night. Additionally, early morning urine was collected after the experimental night (7:00 a.m., Ut1, 4.5 h). In an initial pilot study, GHB (50 mg/kg, Xyrem^®^) or placebo had been administered at 11 p.m. of the experimental night as otherwise described above for the main study. Early morning urine (*n* = 15 volunteers) was collected after the experimental night (7:00 a.m., 8 h, Ut2). All samples were stored at −80 °C until analysis.

### 4.2. Chemicals and Reagents

Commercially available reference standards (listed in the supplementary information) were obtained from Sigma-Aldrich (Buchs, Switzerland) and ReseaChem Life Science (Burgdorf, Switzerland). GHB-glycine was synthesized in-house in cooperation with the Institute of Pharmaceutical Sciences of the ETH Zurich, Switzerland. Acetonitrile (ACN), methanol (MeOH) and water of HPLC grade were purchased from Fluka (Buchs, Switzerland). All other chemicals used were from Merck (Zug, Switzerland) and obtained in the highest grade available.

### 4.3. Sample Preparation

Serum and urine samples collected during and after the experimental nights following placebo and GHB administration were prepared by simple sample dilution and filtration as described in detail elsewhere with slight modifications [13,24].

Serum samples (t0, t1, t2 of 20 participants of the main study) were thawed at room temperature and 75 μL were spiked with 15 μL of the IS mixture (adenosine ribose-D1 15 μmol/L, arginine-13C6, 300 μmol/L, caffeine 3-methyl-13C 200 μmol/L, carnitine trimethyl-D9 100 μmol/L, creatinine N-methyl-D3 500 μmol/L, deoxycholic acid-D4 1.8 μmol/L, D-fructose 13C 120 μmol/L, glycine-13C2 800 μmol/L, glycocholic acid-D4 150 μmol/L, hippuric acid 15N) [24]. 225 μL of a 90/10 *v*/*v* mixture of ice-cold (−20 °C) MeOH:acetone was added and vortexed for 30 s. After centrifugation (14,000 *g*, 10 min), 250 μL of the supernatant was transferred into an autosampler filter vial (0.45 µm PTFE, Thomson Instrument company, Oceanside, CA, USA).

Urine samples (*n* = 19 participants of the main study, Ut1; *n* = 15 participants of the pilot study, Ut2) were thawed at room temperature. After vortexing for 20 s, 200 μL of urine sample were added to 40 μL of the IS mix in autosampler filter vials. Depending on the acquisition method either 200 μL of a 1:1 (*v*/*v*) mixture of eluents A (10 mM ammonium formate with 0.1% (*v*/*v*) formic acid in water) and B (0.1% (*v*/*v*) formic acid in MeOH) or C (25 mM ammonium acetate and 0.1% (*v*/*v*) acetic acid in water) and D (0.1% (*v*/*v*) acetic acid in ACN) was added for measurement on the RP column or HILIC column, respectively. After mixing, the filter vials were carefully closed with a filter plunger and were either analyzed directly as described below or stored at −20 °C until analysis.

Pooled serum and pooled urine samples were prepared by mixing equal volumes of each serum or urine sample (200 µL each) for QC reasons.

### 4.4. UHPLC-HRMS

MS measurements were performed in randomized order in three batches (serum, urine Ut1, urine Ut2) on a Thermo Fischer Ultimate 3000 UHPLC system (Thermo Fischer Scientific, San Jose, CA, USA) coupled to a HR TOF instrument system (TripleTOF 6600, Sciex, Concord, ON, Canada) as described elsewhere and in the supplementary information [13,23,24]. Briefly, two different columns—RP (Waters XSelect HSST RP-C18 column, Waters, Baden-Daettwil, Switzerland) (150 mm × 2.1 mm, 2.5 µm particle size)) and HILIC (Merck SeQuant ZIC HILIC column, Merck, Darmstadt, Germany) (150 mm × 2.1 mm, 3.5 µm particle size)) were used for chromatographic separation. HR MS and MS/MS data were acquired by two methods: TOF MS only and information dependent data acquisition (IDA) separated in positive and negative ionization mode (resolving power 30,000 in MS and 15,000 in MS/MS). A SST described in detail in reference [24] was measured after every fifth sample and was checked for reproducibility of the data by retention time (RT) shifts and peak area comparison using MultiQuant V 2.1 (Sciex, Concord, ON, Canada). Further, a pooled QC sample was additionally measured after every fifth sample.

### 4.5. Data Pre-processing, Normalization

Progenesis QI (Waters Corp., Milford, CT, USA) was used for TOF data only to perform data-preprocessing, alignment, deconvolution, peak picking, initial data normalization and filtering. Data files including MS/MS scans (IDA) were uploaded in the software but used solely for identification. Each run within one batch was automatically matched for similarity to the other runs to pick the run showing highest similarity between runs as a reference for data alignment and normalization. The following peak picking parameters were applied: automatic sensitivity method, sensitivity value 3, no minimum peak width and no retention time limits, ion species [M + H]^+^, [M + 2H]^2+^, [M + H − H_2_O]^+^, [M + NH4]^+^, [M + Na]^+^, [M + 2Na]^2+^, [M − H]^−^, [M − 2H]^−^, [M − H_2_O − H]^−^, [M + Na − 2H]^−^, [M + FA − H]^−^. Automatic deconvolution was performed on features with same retention time and ion mass differences equal to known mass differences between two or more experimental adducts. For (initial) normalization and to compensate for analytical variation, the method in Progenesis Qi previously evaluated as best suited [27] was applied to serum and urine data sets. Briefly, this method calculates a factor multiplied by all ion abundances of all compounds for each sample allowing for recalibration to the picked reference run. A quantitative abundance ratio of all detected ions was calculated between the normalized run and the reference run sample. Urine samples were additionally normalized to the creatinine concentration of each sample, measured by the Jaffe reaction on an Indiko Plus device (Thermo Scientific, Braunschweig, Germany) prior to statistical analysis.

### 4.6. Statistical Analysis/Feature Selection

Data tables for serum, urine t1 and urine t2 exported from Progenesis Qi where filtered by selecting features with an abundance > 500 (counts per s, cps). Different statistical methods were then applied to select compounds potentially changed through GHB consumption. 

Serum and (creatinine-normalized) urine samples at two time-points each were subjected separately to volcano-plot analysis (paired *t*-tests between placebo and GHB session (*p* < 0.05, two-tailed); median fc exceeding 1.5 in at least 70% of sample pairs).

Additionally, mixed effect model calculations taking into account a random effect for the crossover design of the study were performed in R Studio (version 1.2.5033, R Studio, Boston, MA, USA); details are provided in reference [23]. Briefly, the crossover design of the study and the expected inter-day variability, was taken into account through the addition of the baseline level (each feature intensity at t0 after log transformation) as an explanatory variable to the model. Target size (log feature intensity at t1 or t2) was then described through the terms treatment (placebo or GHB), week (in randomized order), interaction (treatment and week), log feature intensity at baseline (t0) and a random effect for each subject.

With an automatic loop, the described test was applied on all features providing a list of *p*-values, whereas only *p*-values ≤ 0.05 were considered to show a significant treatment effect caused by GHB.

### 4.7. Compound Identification

Selected features from statistical analysis were searched on the MS and MS/MS level against an in-house database and against different online databases METLIN [39], the Human Metabolome Database (HMDB, V4.0) [40], NIST 2014 [41] and Lipidblast [42]. If database searches did not provide reliable feature identification, features were tentatively interpreted based on their accurate precursor mass (e.g., [M + H]^+^/[M − H]^−^) and accurate fragment ions calculating their possible molecular composition and respective ppm deviation. The Fragment similarity search tool from METLIN (https://metlin.scripps.edu (accessed on 24 November 2020)) was applied on MS/MS data to identify possible substructures.

Confidence of identification results are indicated as suggested by the MSI [43]: level 1 provides identified compounds and uses two or more measured orthogonal parameters (e.g., RT and mass spectrum) of an authentic chemical standard analyzed under the identical analytical conditions that matches the metabolite present in the sample. Level 2 provides putatively characterized compounds and identification is based on physicochemical properties and/or similarities with mass spectra of public or commercial libraries. Level 3 provides putatively characterized compound classes by spectral similarities to known compounds of a chemical class or based upon physicochemical properties of a chemical class.

## Data Availability

The datasets generated during and/or analyzed during the current study are available from the corresponding author on reasonable request.

## Supplementary Materials

The following are available online at https://www.mdpi.com/2218-1989/11/3/166/s1, Figure S1: QTOF MS/MS spectra used for identification of significantly changed features, Figure S2: QTOF MS/MS spectra of currently still unknown features, Figure S3: Abundance of GHB in urine and serum, Figure S4: QTOF MS/MS spectra of currently still unknown features detected in serum samples, Table S1: Features/feature groups in urine samples significantly changed between placebo and GHB intake, Table S2: Features/feature groups in serum samples significantly changed between placebo and GHB intake, Table S3: Features identified in serum after mixed-effect model statistics; Details on materials and methods.
