# Supplementary material for: Towards Extending the Detection Window of Gamma-Hydroxybutyric Acid—An Untargeted Metabolomics Study in Serum and Urine Following Controlled Administration in Healthy Men
Source: Metabolites. 2021 Mar 12;11(3):166. doi: 10.3390/metabo11030166 (PMC7998200; doi:10.3390/metabo11030166)
Supplement: Supplementary file 1 [file metabolites-11-00166-s001.pdf]

metabolites

Supplementary Material

Extending the Detection Window of Gamma-Hydroxybutyric Acid—An Untargeted Metabolomics Study in Serum and Urine Following Controlled Administration in Healthy Men

Andrea E. Steuer [1,*], Justine Raeber [1], Fabio Simbuerger [1], Dario A. Dornbierer [1,3], Oliver G. Bosch [3], Boris B. Quednow [3,5], Erich Seifritz [3,4,5] and Thomas Kraemer [1]

[1] Department of Forensic Pharmacology & Toxicology, Zurich Institute of Forensic Medicine, University of Zurich, 8057 Zurich, Switzerland; Justine.raeber@bluewin.ch (J.R.); fabiosi@student.ethz.ch (F.S.); dornbierer@pharma.uzh.ch (D.A.D.); Thomas.kraemer@irm.uzh.ch (T.K.)
[2] Department of Psychiatry, Psychotherapy and Psychosomatics, Psychiatric Hospital, University of Zurich, 8032 Zurich, Switzerland; oliver.bosch@bli.uzh.ch (O.G.B.); quednow@bli.uzh.ch (B.B.Q.); erich.seifritz@bli.uzh.ch (E.S.)
[3] Zurich Center for Interdisciplinary Sleep Research (ZiS), University of Zurich, 8091 Zurich, Switzerland
[4] Neuroscience Center Zurich, University of Zurich and Swiss Federal Institute of Technology Zurich, 8057 Zurich, Switzerland
* Correspondence: andrea.steuer@irm.uzh.ch; Tel.: +41-(0)4-4635-5679

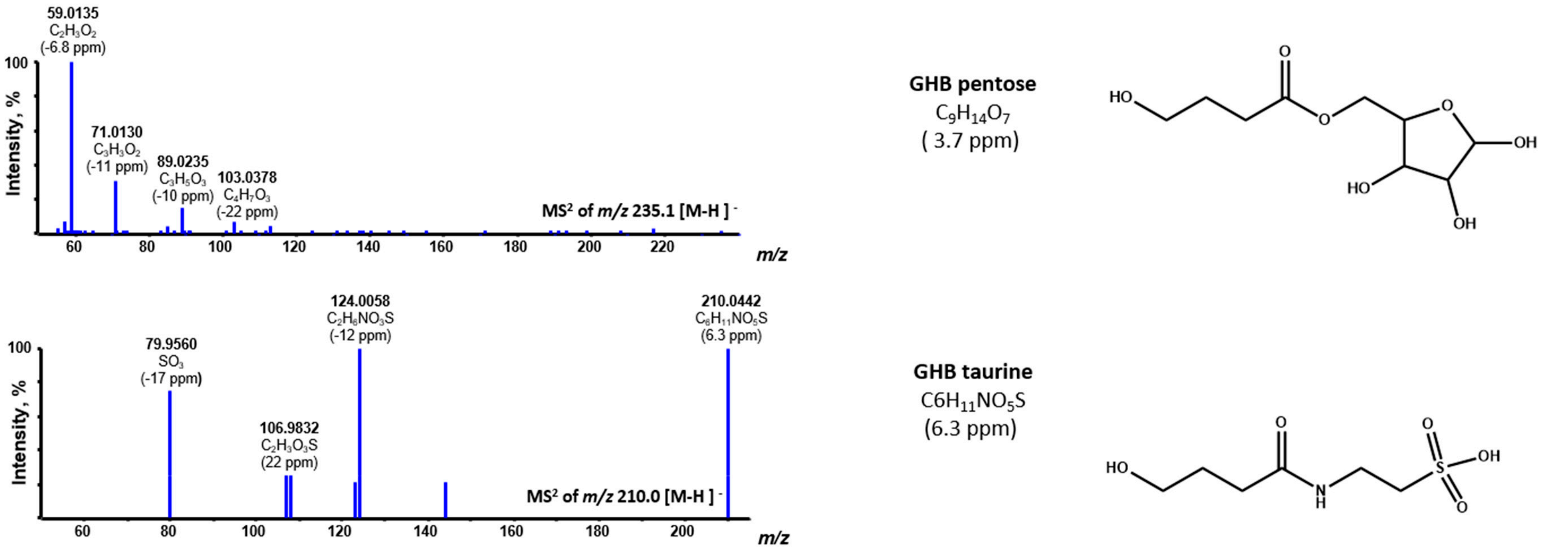
59.0135
$C_2H_3O_2$
(-6.8 ppm)
71.0130
$C_3H_3O_2$
(-11 ppm)
89.0235
$C_3H_5O_3$
(-10 ppm)
103.0378
$C_4H_7O_3$
(-22 ppm)
MS^2 of *m/z* 235.1 [M-H]$^-$
Intensity, %
m/z
GHB pentose
$C_9H_{14}O_7$
(3.7 ppm)
124.0058
$C_2H_6NO_3S$
(-12 ppm)
210.0442
$C_6H_{11}NO_5S$
(6.3 ppm)
79.9560
SO_3
(-17 ppm)
106.9832
$C_2H_3O_3S$
(22 ppm)
MS^2 of *m/z* 210.0 [M-H]$^-$
GHB taurine
C6$H_{11}NO_5S$
(6.3 ppm)

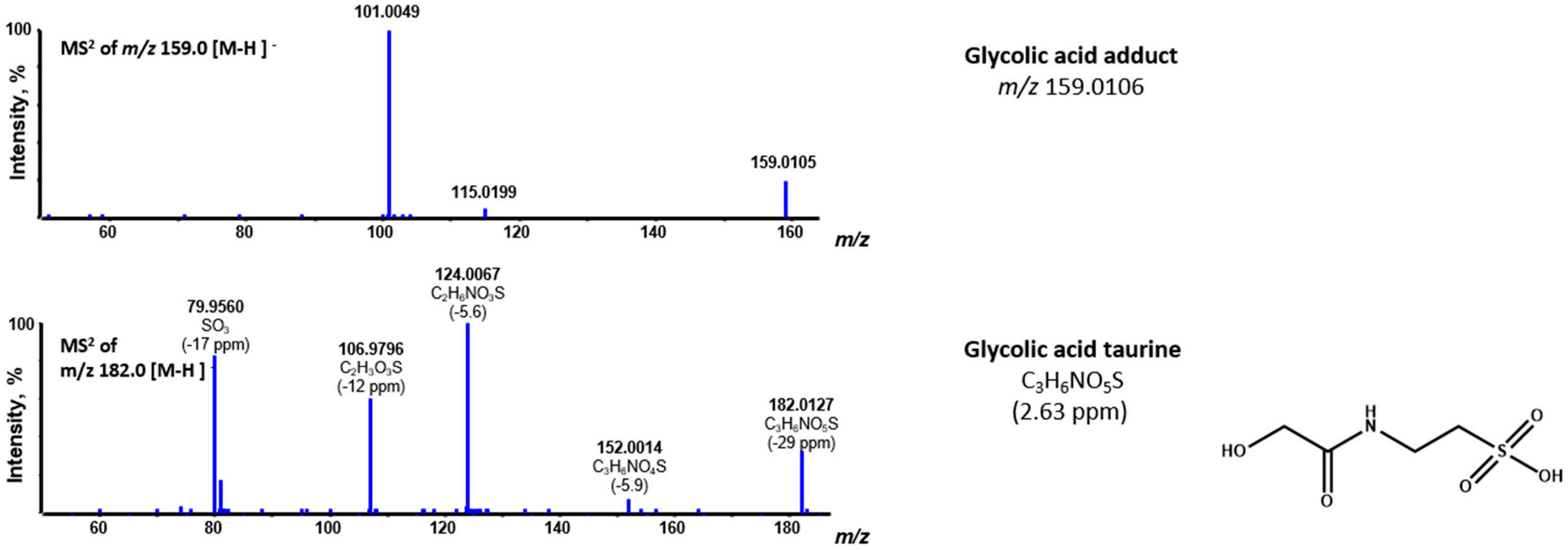

Figure S1. QTOF MS/MS spectra (collision energy 35 eV, collision energy spread ± 15 eV) used for identification of significantly changed features. Given are accurate fragment masses of the respective protonated or deprotonated molecular ions, the calculated sum formula of each fragment, corresponding ppm deviation.

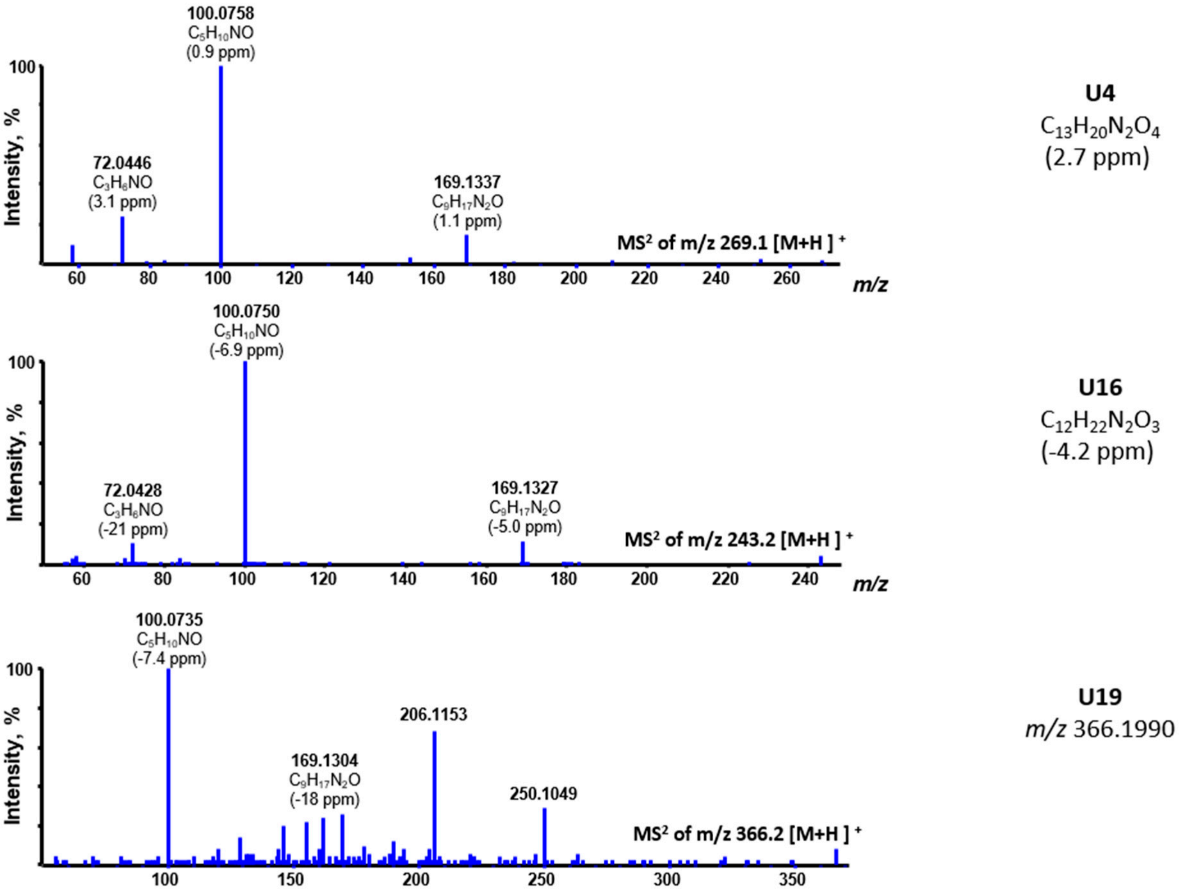
100.0758
C5H10NO
(0.9 ppm)
72.0446
C3H6NO
(3.1 ppm)
169.1337
C9H17N2O
(1.1 ppm)
MS2 of m/z 269.1 [M+H] +
Intensity, %
m/z
U4
C13H20N2O4
(2.7 ppm)
100.0750
C5H10NO
(-6.9 ppm)
72.0428
C3H6NO
(-21 ppm)
169.1327
C9H17N2O
(-5.0 ppm)
MS2 of m/z 243.2 [M+H] +
U16
C12H22N2O3
(-4.2 ppm)
100.0735
C5H10NO
(-7.4 ppm)
206.1153
169.1304
C9H17N2O
(-18 ppm)
250.1049
MS2 of m/z 366.2 [M+H] +
U19
m/z 366.1990

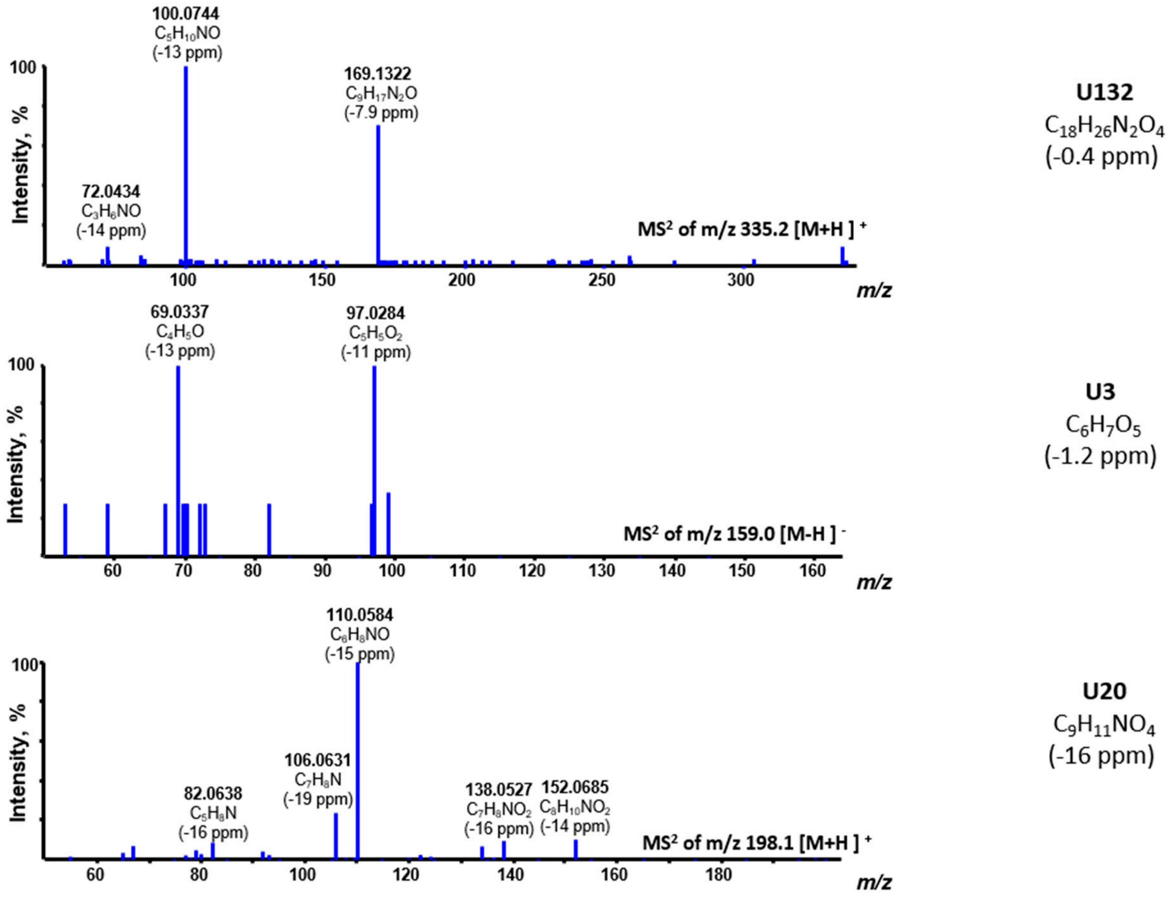

Figure S2. QTOF MS/MS spectra (collision energy 35 eV, collision energy spread ± 15 eV) of currently still unknown features. Given are accurate fragment masses of the respective protonated or deprotonated molecular ions, the calculated sum formula of each fragment (if available) and corresponding ppm deviation.

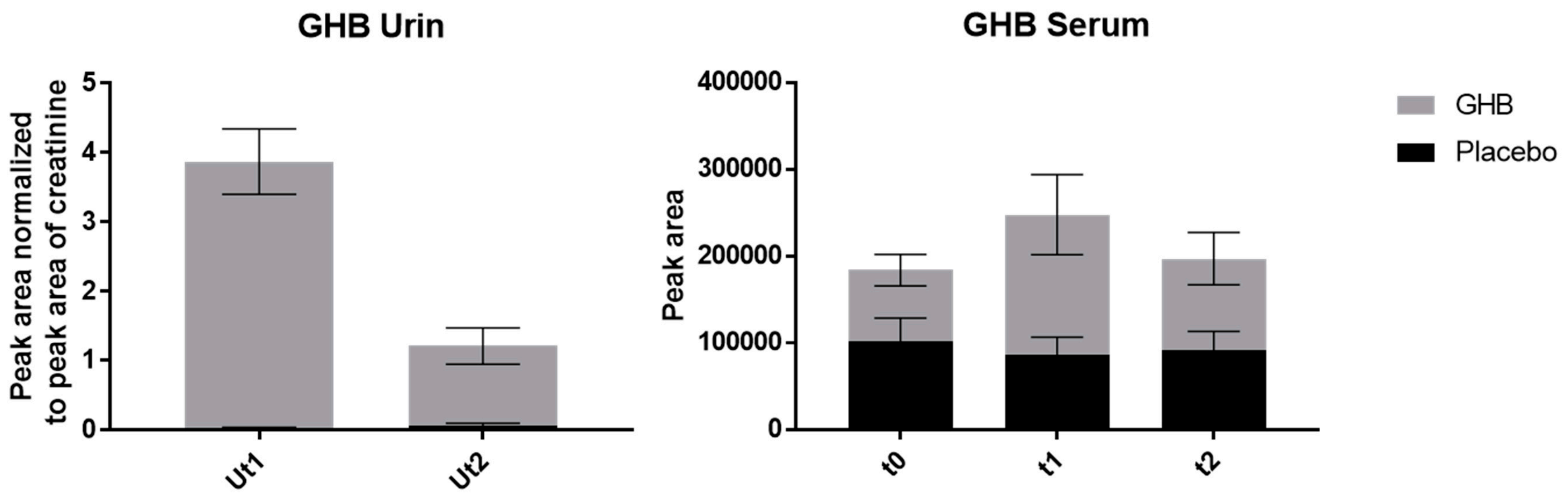

Figure S3. Abundance of GHB in urine (left panel) and serum (right panel). Depicted are urinary analyte peak area to creatinine ratios for placebo (black) and GHB group (grey) at Ut1 (n = 19 each) and Ut2 (n = 15 each) and serum peak areas of GHB before (t0) and 4.5 h (t1) and 16.5 h after intake (t2). Data points represent mean and SEM of replicate measurements.

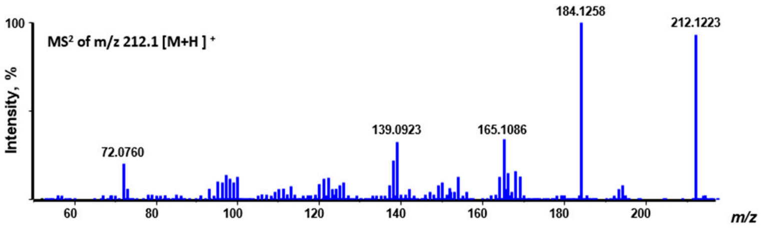

S2
m/z 212.1223

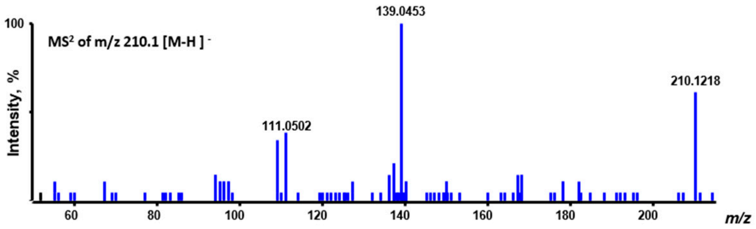

S2
m/z 210.1079

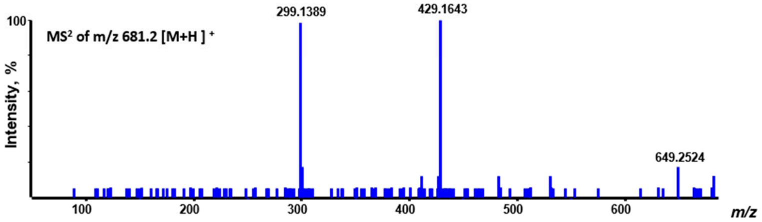

S32
m/z 681.2452

Figure S4. QTOF MS/MS spectra (collision energy 35 eV, collision energy spread ± 15 eV) of currently still unknown features detected in serum samples. Given are accurate fragment masses of the respective protonated or deprotonated molecular ions.

Table S1. Features/feature groups in urine samples selected based on significant changes and fold-changes > 1.5 between placebo and GHB intake, sorted by highest significance (p-value). Statistical comparison was performed by paired t-tests (< 0.05) and median fold-change filtering (> 1.5). Identification confidence was assigned based on the Metabolomics Standard Initiative (MSI) as follows: confirmation using MS/MS information and co-elution with authentic standards (level 1); confirmation through comparison of experimental MS/MS spectra with online databases (level 2); and annotation to putatively characterized compound classes (level 3). RT, retention time; m/z mass to charge ratio; p 4.5/8 p-value at time-point 4.5h and 8h, respectively; FC foldchange; HILIC hydrophilic interaction liquid chromatography; RP reversed phase.

	**Compound**	**Method**	**RT (min)**	***m/z***	**Fragment Ions**	**Formula**	***p* 4.5**	**FC 4.5**	***p* 8**	**FC 8**	**(tentative) Identification**	**.**	**Adduct**
U1	3.99_235.0819m/z	RP -	3.99	235.0819	59, 71, 89, 103, 199, 217	C9H14O7	0.000	410	0.001	46	GHB-pentose	3	M-H
	4.11_235.0829 m/z	HILIC -	4.11	235.0829	59, 71, 89, 103, 198	C9H14O7	0.000	83					
U2	7.18_232.0830m/z	HILIC -	7.18	232.0830	102, 128, 146	C9H15NO6	0.000	666			GHB-glutamate	3	M-H
U3	1.72_159.0295m/z	RP -	1.72	159.0294	69, 97, 99	C6H7O5	0.000	26					
U4	4.17_267.1350m/z	RP -	4.17	267.1350	99, 135, 138, 225, 267	C13H18N2O4	0.000	4.6	0.000	24			
	4.92_267.1357m/z	HILIC -	4.92	267.1356	99, 135, 138, 225, 267	C13H18N2O4	0.000	7.9	0.000	21			
	4.18_268.1419n	RP +	4.18	269.1496	72, 100, 169, 252	C13H20N2O4	0.000	4.3	0.000	7.6			
	4.82_115.0574n	HILIC +	4.82	269.0857	72, 100, 137, 156, 169, 269	C13H20N2O4			0.004	376			
U5	1.70_315.0697m/z	RP -	1.70	315.0697	no MS/MS		0.000	23					
	1.70_405.1148m/z	RP -	1.70	405.1147	no MS/MS		0.000	22					
	1.70_455.1408m/z	RP -	1.70	455.1408	no MS/MS		0.000	10					
U6	4.63_210.0442m/z	HILIC -	4.63	210.0441	80, 107, 124	C6H11NO5S	0.000	18			GHB-taurine	3	M-H
	4.67_539.2039m/z	HILIC -	4.67	539.2038	210, 539		0.000	10			GHB-taurine adduct +327	3	
U7	4.58_340.1233n	RP +	4.58	363.1161	85, 109, 123, 145, 155, 286, 363	C13H14N8O5	0.000	12					
	4.57_340.1262n	RP -	4.57	385.1251	no MS/MS		0.000	9.9					
U8	7.32_183.0301m/z	HILIC -	7.32	183.0301	81, 101, 121, 183	C5H2N4O2S	0.000	2.1					
U9	5.70_160.0620m/z	HILIC -	5.70	160.0620	74, 86	C6H9NO4	0.000	490			GHB-glycine	1	M-H
U10	4.58_402.1151m/z	RP -	4.58	402.1150	no MS/MS		0.000	21					
U11	0.92_159.0105m/z	RP -	0.92	159.0105	71, 88, 101, 115, 159		0.000	5.0	0.011	2.4	Glycolic acid adduct	1	M+FA-H

U12	1.10_226.0883n	RP -	1.10	247.063	57, 101, 145, 203, 247		0.000	2.9	0.003	2.8			
	1.08_279.0348m/z	RP -	1.08	279.0348	87, 101, 113, 145, 171, 202, 215, 264				0.012	2.0			
U13	4.18_352.1127m/z	RP -	4.18	352.1127	no MS/MS		0.000	4.2					
	4.17_367.0227m/z	RP -	4.17	367.0227	no MS/MS		0.000	9.7					
	4.18_557.2596m/z	RP -	4.18	557.2596	no MS/MS		0.000	12					
U14	5.33_218.9970m/z	HILIC -	5.33	218.9970	135, 161, 175, 219	C7H6O6S	0.004	4.6					
	5.33_237.0079m/z	HILIC -	5.33	237.0079	77, 119, 135, 161, 175, 219	C7H8O7S	0.000	4.6					
	5.32_295.0119m/z	HILIC -	5.32	295.0118	no MS/MS		0.012	4.4					
	5.36_314.9853m/z	HILIC -	5.36	314.9853	75, 124, 135, 161, 219, 239, 279		0.000	3.8					
U15	2.15_87.0436m/z	RP +	2.15	87.0436	69, 87		0.000	45					
	1.71_244.0075m/z	RP -	1.71	244.0075	no MS/MS		0.000	92					
	1.71_341.0486m/z	RP -	1.71	341.0486	no MS/MS		0.000	150					
	1.71_403.0975m/z	RP -	1.71	403.0975	no MS/MS		0.000	2.1					
U16	6.95_241.0355m/z	HILIC -	6.95	241.0355	no MS/MS		0.000	480					
	6.92_243.1688m/z	HILIC +	6.92	243.1687	no MS/MS	C12H22N2O3	0.000	7.6	0.004	6.0			
	2.58_243.1699m/z	RP +	2.58	243.1699	72, 100, 169, 243	C12H22N2O3	0.001	8.5					
U17	3.46_143.0346m/z	RP -	3.46	143.0346	59	C6H8O4	0.000	3.2					
	1.70_143.0349m/z	HILIC -	1.70	143.0349	57, 59, 99, 143	C6H6O4	0.000	3.5	0.006	7.5	Hydroxyadipic acid lactone	2	M-H
U18	7.27_248.1487m/z	HILIC +	7.27	248.1487	57, 60, 85, 87, 103, 144, 248	C11H21NO5	0.000	150	0.003	18	GHB-carnitine	1	M+H
	2.35_248.1496m/z	RP +	2.35	248.1496	57, 60, 85, 87, 103, 144, 248	C11H21NO5	0.000	76	0.001	25	GHB-carnitine	1	M+H
U19	5.65_366.2017m/z	RP +	5.65	366.2016	100, 129, 146, 169, 206, 250, 366	C18H27N3O5	0.000	14	0.010	55			
	5.42_366.2020m/z	RP +	5.42	366.2020		C18H27N3O5	0.000	3.1	0.001	24			
U20	5.89_152.0692m/z	RP +	5.89	152.0691	65, 67, 82, 93, 106, 110, 134, 152	C8H9NO2			0.000	140			
	5.89_197.0676n	RP +	5.89	198.0738	65, 67, 82, 93, 106, 110, 134, 152	C9H11NO4			0.000	18			
U21	4.13_254.1016m/z	HILIC +	1.00	254.1016	94, 122, 137, 179	C12H15NO5	0.000	3.9					

U22	2.08_104.0472n	HILIC -	2.08	103.0398	55, 73	C4H6O3	0.000	8.2			GHB	1	M-H
U23	4.94_182.0133m/z	HILIC -	4.94	182.0133	80, 81, 107, 124, 152, 182	C3H6NO5S	0.000	1.8			Glycolic acid taurine	3	M-H
U24	4.95_174.0772m/z	HILIC -	4.95	174.0771	58, 86, 100, 128, 138	C7H11NO4	0.000	3.0					
U25	4.35_414.1393m/z	RP +	4.35	414.1392	121, 167				0.000	12			
U26	6.69_319.9906m/z	HILIC -	6.69	319.9906	59, 111, 157, 201		0.000	19					
U27	1.81_228.0858m/z	RP +	1.81	228.0857	79, 96, 136, 182, 228	C10H13NO5	0.000	5.7					
U28	5.57_325.0479n	HILIC -	5.57	306.0300	no MS/MS		0.000	1.9					
	5.57_339.9810m/z	HILIC -	5.57	339.9810	82, 126, 147, 195, 213, 260, 304		0.011	2.6					
U29	4.92_274.1646m/z	RP +	4.92	274.1646	57, 85, 113, 144, 215, 274	C13H23NO5	0.000	2.2					
U30	3.84_250.1089m/z	HILIC -	3.84	250.1088	no MS/MS		0.000	10					
	3.85_435.2589m/z	HILIC -	3.85	435.2589	no MS/MS		0.050	2.0	0.016	2.0			
U31	3.06_288.1436m/z	RP +	3.06	288.1436	no MS/MS		0.005	4.9					
	2.80_288.1410m/z	RP +	2.80	288.1409	60, 85, 110, 116, 127, 145, 288	C8H21N3O8	0.002	6.6	0.000	18			
U32	5.56_226.0737m/z	HILIC -	5.56	226.0737	71, 99, 125, 138, 151	C10H11NO5	0.001	2.1	0.001	5.9			
	5.52_99.0077m/z	HILIC -	5.52	99.0077	71				0.001	2.7			
U33	5.44_375.0064m/z	HILIC -	5.44	375.0064	85, 113, 135, 203, 237, 281, 339		0.001	3.9					
	5.46_383.1330m/z	HILIC -	5.46	383.1330	59, 99, 113, 123, 207, 383		0.014	1.5					
U34	7.33_633.2081n	HILIC +	7.33	675.2411	138, 204, 292, 366, 657		0.001	1.5					
U35	0.93_233.0473m/z	RP -	0.93	233.0472	75, 101, 121, 233	C9H12O5S	0.001	1.8					
U36	5.36_192.9817m/z	HILIC -	5.36	192.9817	77, 91, 135, 193	C4H5N2O3PS	0.001	14	0.035	2.7			
U37	4.63_129.0194m/z	HILIC -	4.63	129.0194	57, 85, 129	C5H6O4	0.041	2.3	0.001	4.9	HO-butenoic acid lactone	2	M+FA-H

U38	5.48_270.9593m/z	HILIC -	5.48	270.9593	77, 135, 137, 195, 209, 253	C8H3N2O5PS	0.041	17		
	5.36_270.9596m/z	HILIC -	5.36	270.9596	77, 135, 137, 195, 209, 253	C8H3N2O5PS	0.001	9.9	0.041	6.4
U39	4.00_246.0432m/z	HILIC -	4.00	246.0432	73, 84, 117, 162, 246				0.001	11
	4.00_342.0301m/z	HILIC -	4.00	342.0301	80, 95, 117, 162, 178				0.018	17
U40	0.92_263.0577m/z	RP -	0.92	263.0577	79, 115, 143, 173	C10H14O6S	0.001	1.8		
U41	10.43_642.2668m/	RP -	10.43	642.2668	113, 181, 291		0.022	2.8		
	4.54_642.2674m/z	HILIC -	4.54	642.2674	113, 181, 291		0.001	3.3		
U42	2.13_196.0573n	RP -	2.13	217.0320			0.002	2.4		
U43	13.30_593.3275m/z	RP +	13.30	593.3275					0.002	1.5
	13.43_593.3285m/z	RP +	13.43	593.3285	124, 180, 303, 344, 468, 593				0.031	3.5
	1.15_593.3306m/z	HILIC +	1.15	593.3305	124, 180, 303, 344, 468, 593				0.010	26
	13.43_595.3431m/z	RP +	13.43	595.3430	180, 303, 470, 595				0.016	5.3
U44	6.73_282.1856m/z	HILIC +	6.73	282.1856	150	C11H15N5O4			0.002	2.6
U47	4.47_238.0928n	HILIC +	4.47	221.0894	91, 130, 157	C11H12N2O3	0.002	2.4		
U48	11.20_558.2866m/z	RP +	11.20	558.2866	149, 311, 329, 347,	365, 558			0.002	1.6
U49	1.17_632.1812m/z	HILIC -	1.17	632.1811	108, 188, 268, 443, 632				0.003	1.7
U50	7.00_227.0562m/z	HILIC -	7.00	227.0561			0.003	2.6		
U51	5.35_250.9870m/z	HILIC -		250.9870			0.003	7.9		
U52	4.77_541.2631m/z	HILIC -	4.77	541.2630	113, 175, 301, 335, 415, 481				0.009	1.6
	11.60_541.2647m/	RP -	11.60	541.2646	113, 175, 301, 335, 415, 481				0.008	1.5
	10.86_541.2649m/	RP -	10.86	541.2648	113, 175, 301, 335, 415, 481				0.003	1.5
	11.48_542.2680n	RP +	11.48	543.2754	141, 253, 271, 331, 349, 489				0.007	1.6

U53	4.65_361.1104m/z	HILIC -	4.65	361.1103			0.003	2.5				
U54	6.67_228.1449n	HILIC -	6.67	265.0934		C10H17O8	0.004	2.8				
U55	4.12_292.0396n	HILIC -	4.12	273.0217	51, 99, 134, 149, 178, 193, 229				0.004	3.4		
U56	2.11_183.0147m/z	RP -	2.11	183.0147	69, 97, 113	C5H2N4O4			0.004	1.9		
U57	4.67_659.2692n	HILIC -	4.67	640.2513	311, 445, 640		0.005	2.9				
U58	3.61_240.1083m/z	RP +	3.61	240.1082	56, 96, 109, 165, 168, 195	C9H13N5O3	0.006	1.9				
U59	5.66_389.0685m/z	HILIC -	5.66	389.0685	85, 113, 135, 175, 274, 389		0.006	47				
U60	7.34_205.0624m/z	HILIC -	7.34	205.0624			0.009	33				
	7.35_249.0526m/z	HILIC -	7.35	249.0525	67, 93, 117, 125, 133, 146, 190, 205	C11H8N2O5	0.007	13				
U61	4.31_401.1790m/z	HILIC +	4.31	401.1789			0.007	2.6				
U62	3.11_363.1758n	HILIC +	3.11	364.1838					0.008	4.0		
U63	7.37_307.0561m/z	HILIC -	7.37	307.0561	221, 265	C12H21NO8	0.008	2.8				
U64	4.49_331.2239m/z	HILIC +	4.49	331.2238	99, 155, 253				0.009	1.6		
U65	4.74_283.1605m/z	RP +	4.74	283.1604	110, 151				0.009	2.0		
U66	5.89_205.0341m/z	HILIC -	5.89	205.0341	53, 73, 99, 125, 167	C7H8O7 / C8H4N4O3			0.009	2.3		
U67	5.51_350.8976m/z	HILIC -	5.51	350.8976	75, 101, 211, 274, 332	C9H4O7P2S2	0.009	2.7				
U68	4.96_322.0697m/z	HILIC -	4.96	322.0696	79, 124, 131, 146, 153, 171, 197, 322		0.010	2.1				
U69	0.86_427.1763m/z	HILIC -	0.86	427.1762	97, 245, 427				0.010	1.9		
U70	1.33_111.0077m/z	RP -	1.33	111.0076	67.00000	C5H2O3			0.024	1.5		
	1.33_191.0205m/z	RP -	1.33	191.0204	67, 85, 87, 111, 129	C6H6O7			0.017	1.5	Citric acid	1
	1.32_289.0377m/z	RP -	1.32	289.0377	57, 67, 111, 155, 217, 289				0.011	1.8		
	1.33_293.9966m/z	RP -	1.33	293.9965	67, 111, 129, 136, 154				0.036	1.6		
	1.33_294.9935m/z	RP -	1.33	294.9934	67, 85, 111, 154, 199, 295				0.033	1.5		

U71	9.36_198.0013m/z	RP +	9.36	198.0013	77, 96, 108, 135, 136, 151, 180	C7H3NO6			0.011	1.6			
U72	2.40_267.1097m/z	RP +	2.40	267.1097	96, 251		0.011	2.2					
U73	7.23_275.1701m/z	HILIC +	7.23	275.1701	60, 85, 114, 157, 275	C12H22N2O5	0.012	2.5					
U74	5.09_218.1032m/z	RP -	5.09	218.1032	71, 88, 99, 146	C9H15NO5			0.013	1.5			
U75	7.27_169.1320m/z	HILIC +	7.27	169.1319	58, 72, 95, 100, 123	C9H17N2O			0.013	2.3			
U76	4.49_564.1761m/z	HILIC -	4.49	564.1761			0.015	3.3					
U77	8.60_260.9993m/z	HILIC -	8.60	260.9993	87, 159, 199		0.015	1.6					
U78	1.52_165.0659m/z	HILIC +	1.52	165.0658	65, 105, 123, 150	C8H8N2O2	0.015	1.9					
U79	7.34_118.0598m/z	HILIC +	7.34	118.0597	55, 58, 59, 72, 76	C3H7N3O2	0.016	1.6					
U80	1.09_237.0875n	RP -	1.09	272.0569	82, 100, 126, 272				0.016	1.6			
U81	8.61_262.1283m/z	HILIC +		262.1283	60, 85, 103, 144, 262	C11H19NO6	0.016	1.3			Succinylcarnitine	2	M+H
U82	0.60_143.0147m/z	HILIC -	0.60	143.0147					0.044	8.5			
	0.60_222.9730m/z	HILIC -	0.60	222.9729	59, 80, 109, 143, 223	C6H6O5S2			0.016	2.6			
U83	10.99_367.2337n	RP +	10.99	332.2382	85, 144, 153, 255, 332	C17H33NO5			0.016	1.6			
U84	1.07_119.0346m/z	RP -	1.07	119.0345	57, 75	C4H6O4	0.017	1.6			Dihydroxybutyric acid	2	M-H
U85	4.09_387.0770m/z	HILIC -	4.09	387.0769	113, 211, 387		0.018	3.1					
U86	11.49_254.1138n	RP +	11.49	255.1207	55, 93, 135, 149, 195				0.018	1.8			
U87	5.85_246.1486n	HILIC +	5.85	310.1643	60, 85, 121, 144, 149, 167, 251	C16H23NO5			0.018	1.7			
U88	11.01_213.1122m/	RP -	11.01	213.1122	57, 97, 133, 151, 169, 195	C11H16O4			0.018	1.7			
U89	9.85_238.1537m/z	HILIC +	9.85	238.1536	56, 84, 119, 137, 193				0.018	2.2			
U90	5.08_402.1383m/z	RP +	5.08	402.1383	103, 149, 209, 273, 367		0.019	1.6					
	6.34_384.1050n	RP +	6.34	402.1386			0.026	3.4					
U91	5.51_251.1940n	RP +	5.51	290.1571	60, 85, 101, 129, 144, 185, 231, 290	C13H23NO6			0.021	1.7			
	5.89_251.1785n	HILIC +	5.89	290.1596	60, 85, 101, 129, 144, 185, 231, 290	C13H23NO6			0.019	2.3			
U92	1.74_94.0033n	HILIC +	1.74	136.0386	53, 67, 80, 92, 108, 136				0.020	1.5			
	1.64_153.0655m/z	HILIC +	1.64	153.0654	53, 78, 92, 108, 110, 122, 136, 153	C7H8N2O2			0.031	1.5			
U93	1.07_291.0894m/z	RP -	1.07	291.0894			0.020	1.7					

U94	14.33_408.2849n	RP +	14.33	426.3187	213, 245, 337, 355, 373, 426				0.020	1.6	Cholic acid	1	M+NH4
U95	4.90_326.0603n	RP +	4.90	327.0675	99, 151, 175, 285, 327	C8H15N4O8P	0.022	2.1					
U96	8.47_270.0967n	HILIC -	8.47	269.0896	96, 110, 113, 154	C10H12N4O5	0.022	1.7			Asp-His/ His-Asp	2	M-H
	8.62_271.1025m/z	HILIC +	8.62	271.1025	74, 95, 110, 156, 164, 208, 254	C10H14N4O5	0.028	1.5			Asp-His/ His-Asp	2	M+H
U97	1.49_291.0510m/z	HILIC -	1.49	291.0510					0.022	1.7			
U98	4.35_206.9967m/z	RP -	4.35	206.9966	71, 73, 80, 99, 127, 207	C6H6O6S / C5H7N2O3PS	0.048	1.5					
	4.35_437.9894n	RP -	4.35	436.9827			0.023	2.1					
U99	3.47_376.1454m/z	HILIC -	3.47	376.1454			0.023	3.3					
U100	8.39_162.0873n	HILIC +	8.39	388.1233					0.023	2.1			
U101	5.45_495.1189m/z	HILIC -	5.45	495.1188	495.00000				0.046	2.3			
	5.47_495.1489m/z	HILIC -	5.47	495.1488	85, 113, 175, 241, 256, 271, 477				0.023	2.9			
U102	0.70_231.0121m/z	HILIC -	0.70	231.0121	92, 136, 151				0.024	2.7			
U103	4.60_381.0131m/z	HILIC -	4.60	381.0131			0.024	2.7					
U104	6.99_250.0283n	HILIC +	6.99	523.0443	148, 273, 398		0.047	2.2					
	6.98_626.0776m/z	HILIC +	6.98	626.0776	126, 251, 376		0.024	4.5					
	6.99_773.0740m/z	HILIC +	6.99	773.0740	148, 273, 398, 523, 648, 773		0.033	2.3					
U105	4.79_258.0630n	HILIC -	4.79	293.0467	80, 119, 150, 165, 205, 221, 293				0.024	3.3			
U106	1.26_236.0740n	RP -	1.26	271.0433			0.024	4.9					
U107	10.47_424.1902m/	RP -	10.47	424.1901	120.00000		0.025	1.8					
U109	10.92_359.2616n	RP +	10.92	398.2494	85, 131, 183, 219, 321, 363, 398				0.026	1.6			
U110	3.28_501.2320m/z	RP -	3.28	501.2320			0.026	8.0					

U111	1.51_141.0642m/z	RP +	1.51	141.0642	54, 68, 81, 95, 123	C6H8N2O2			0.026	1.8
U112	4.36_453.9632n	RP -	4.36	452.9559			0.027	1.8		
U113	4.76_475.0783m/z	HILIC -	4.76	475.0783			0.027	1.7		
U114	6.97_385.0420m/z	HILIC -	6.97	385.0420	59, 147, 191, 260, 325		0.027	1.9		
U115	1.64_320.1540m/z	HILIC -	1.64	320.1539	80, 124, 180, 225, 320		0.028	1.8		
U116	1.41_417.2081m/z	HILIC -	1.41	417.2080			0.028	1.6		
U117	5.40_244.1866n	HILIC +	5.40	286.2002	60, 85, 227, 286	C15H27NO4			0.028	1.7
U118	6.15_364.9483m/z	RP +	6.15	364.9483	95, 203, 285, 365		0.030	1.7		
U119	6.52_371.0956m/z	HILIC -	6.52	371.0955	85, 113, 136, 195, 291, 371				0.031	2.0
U120	1.25_445.1878m/z	HILIC -	1.25	445.1877	97, 385, 415, 445				0.031	1.6
U121	1.08_381.0267m/z	HILIC -	1.08	381.0267	164, 229, 286, 301, 381				0.031	1.5
U122	5.86_553.2904m/z	HILIC +	5.86	553.2903	85, 130, 229, 331, 424, 553				0.031	1.6
U123	0.71_125.0247m/z	HILIC -	0.71	125.0246	55, 63, 69, 81, 125	C6H4O3	0.032	1.6		
U124	2.40_146.0801m/z	RP +	2.40	146.0801	82, 84, 128, 146	C6H11NO3			0.032	1.6
U125	1.78_229.0710m/z	HILIC -	1.78	229.0709	73, 99, 121, 149, 229		0.033	2.9		
U126	3.21_271.1915m/z	HILIC -	3.21	271.1914			0.033	6.6		
U127	4.57_283.1631m/z	HILIC +	4.57	283.1630	58, 100, 142	C16H30N2O2			0.033	3.7
U128	7.75_475.2028m/z	HILIC -	7.75	475.2028	154, 208, 266, 378, 457, 475				0.036	1.7

U129	3.32_473.2015m/z	HILIC -	3.32	473.2014			0.036	17					
U130	6.69_355.1035m/z	RP -	6.69	355.1035	75, 135		0.037	2.0					
U131	10.72_321.1326m/z	RP +	10.72	321.1325	83, 108, 136, 150, 168, 321	C12H24N4O6 / C13H20N8O2	0.037	1.6					
U132	6.44_370.2099n	RP +	6.44	335.1960	72, 100, 169, 259, 335	C18H26N2O4	0.037	2.5					
U133	3.56_303.0182m/z	HILIC -	3.56	303.0181	123, 161, 179, 223, 303		0.038	5.7					
	3.56_306.1019m/z	HILIC -	3.56	306.1019			0.037	3.4					
U134	3.77_185.0915m/z	RP +	3.77	185.0914	60, 70, 98, 139, 185	C8H12N2O3	0.038	1.6					
U135	9.07_255.1324m/z	HILIC +	9.07	255.1323	56, 84, 108, 130, 139, 219, 255		0.039	1.5					
	5.18_254.1259n	RP +	5.18	255.1331	84, 122, 150, 168, 213, 255	C12H18N2O4	0.046	1.8					
U136	6.14_355.0096m/z	RP -	6.14	355.0095			0.040	2.5					
U137	3.67_218.1371m/z	RP +	3.67	218.1370	60, 85, 144, 159, 218	C10H19NO4			0.041	2.1	Propionylcarnitine	1	M+H
U138	1.04_429.1922m/z	HILIC -	1.04	429.1921	97, 429				0.042	1.6			
U139	1.24_203.9974m/z	HILIC -	1.24	203.9973	80, 82, 124	C6H7NO5S	0.042	2.0					
U140	3.89_299.1457n	RP +	3.89	300.1530	95, 112, 168, 300				0.042	3.3			
U141	8.53_356.1211m/z	HILIC -	8.53	356.1211	149, 154, 229, 321		0.042	1.6					
U142	1.24_319.0129m/z	HILIC -	1.24	319.0129	81, 113, 167, 192, 239, 319		0.044	1.5					
U143	1.21_303.0530m/z	RP -	1.21	303.0529	83, 101, 111, 127, 145				0.044	1.5			
U144	0.97_377.1605m/z	HILIC -	0.97	377.1605	80, 108, 188, 377		0.045	3.9					

U145	8.51_304.2103m/z	RP +	8.51	304.2103	60, 85, 125, 145, 227, 304	C15H29NO5			0.045	1.9			
U146	0.67_499.0886m/z	HILIC +	0.67	499.0886	142, 358, 389, 419, 499		0.045	1.9					
U148	4.56_327.0727m/z	HILIC -	4.56	327.0726	99, 151, 327		0.047	2.4					
U149	8.87_130.0639m/z	RP +	8.87	130.0639	51, 77, 103, 130	C4H7N3O2			0.047	1.5			
	8.87_176.0694m/z	RP +	8.87	176.0694	51, 77, 103, 130	C5H9N3O4			0.047	1.5			
U150	8.78_328.1233m/z	HILIC +	8.78	328.1232			0.047	1.5					
U151	6.09_263.1721n	RP +	6.09	246.1688	60, 85, 187, 246	C12H23NO4			0.047	1.7	2-Methylbutyroxylcarnitine	2	M+H
U152	0.58_259.0272m/z	HILIC -	0.58	259.0271	92, 136, 179, 259	C6H6N6O4S			0.047	1.7			
U153	2.11_722.5014m/z	HILIC +	2.11	722.5013					0.047	2.0			
U154	10.18_431.1829m/z	RP -	10.18	431.1829	113, 134, 148, 174, 259, 273, 291, 299, 345, 431				0.048	2.7			
U155	6.56_348.0997n	HILIC +	6.56	390.1357	122, 137, 179, 197, 390				0.048	3.9			
U156	6.59_413.1101m/z	HILIC -	6.59	413.1101	99, 161, 195, 413		0.048	1.9					
U157	1.69_476.3043m/z	HILIC +	1.69	476.3042	89, 133, 177, 221, 459				0.048	2.2			
U158	1.20_157.0855m/z	HILIC -	1.20	157.0855	83, 98				0.048	1.7			
U159	7.38_301.0034m/z	HILIC -	7.38	301.0033	59, 99, 111, 157, 173, 229, 301		0.049	20					
U160	0.92_75.0084m/z	RP -	0.92	75.0084			0.075	1.8			Glycolic acid	1	M-H

Table S2. Features/feature groups in serum samples selected based on significant changes and fold-changes > 1.5 between placebo and GHB intake, sorted by highest significance (*p*-value). Statistical comparison was performed by paired t-tests (<0.05) and median fold-change filtering (>1.5). Identification confidence was assigned based on the Metabolomics Standard Initiative (MSI) as follows: confirmation using MS/MS information and co-elution with authentic standards (level 1); confirmation through comparison of experimental MS/MS spectra with online databases (level 2); and annotation to putatively characterized compound classes (level 3). RT, retention time; m/z mass to charge ratio; *p* 4.5/16.5 *p*-value at time-point 4.5 h and 16.5 h, respectively; FC foldchange; HILIC hydrophilic interaction liquid chromatography; RP reversed phase.

	Compound	Method	RT (min)	*m/z*	Fragment Ions	Formula	*p* 4.5	FC 4.5	*p* 16.5	FC 16.5	Final Identification	.	Adducts
S1	5.37_269.1509n	HILIC -	5.37	268.1436	154, 192		0.001	−1.6	0.005	−1.4			
	5.65_268.1440m/z	HILIC -	5.65	268.1439	154, 192		0.059	−1.8	0.000	−2.2			
	1.99_210.1080m/z	RP -	1.99	210.1079	111, 139, 210	C7H13N7O	0.023	−2.0	0.164	−1.7			
	6.28_210.1097m/z	HILIC -	6.28	210.1096	109, 139, 167	C7H13N7O	0.025	−13.1	0.032	−20.8			
S2	6.46_210.1106m/z	HILIC -	6.46	210.1106	109, 139, 167	C7H13N7O	0.001	−68.2	0.005	−74.1			
	1.96_212.1223m/z	RP +	1.96	212.1223	72, 139, 165, 184	C7H14N7O	0.001	−6.8	0.010	−7.8			
	1.87_278.0953m/z	RP -	1.87	278.0952	111, 139, 182, 210		0.007	−4.7	0.008	−10.2			
	1.98_295.0864m/z	RP -	1.98	295.0863	111, 139, 210		0.026	−2.3	0.141	−1.9			
S3	5.86_246.1699m/z	HILIC +	5.86	246.1699	60, 85, 187, 246	C12H23NO4	0.001	−1.7	0.889	−1.1	2-Methylbutyroxylcarnitine	2	M+H
S4	4.00_157.0797m/z	RP +	4.00	157.0797	53, 84, 111, 123, 139	C10H8N2	0.004	2.3	0.022	2.3			
S5	3.80_647.2394m/z	HILIC -	3.80	647.2393	219, 383, 427, 647		0.012	−1.5	0.722	−1.1			
	3.81_679.2634m/z	HILIC -	3.81	679.2633	219, 383, 427, 647		0.008	−2.0	0.702	−0.9			
S6	0.79_165.0413m/z	RP -	0.79	165.0413	75		0.009	1.6	0.022	2.1	Dihydroxybutyric acid	2	M+FA-H
S7	0.63_411.2391m/z	HILIC -	0.63	411.2391	97		0.010	1.5	0.084	−1.1			
S8	1.31_185.1181m/z	HILIC -	1.31	185.1180	no MS/MS		0.011	1.7	0.218	−0.6			
S9	0.84_386.2339m/z	HILIC -	0.84	386.2339	no MS/MS		0.012	1.6	0.566	−1.0			
S10	2.84_279.6116m/z	HILIC -	2.84	279.6116	no MS/MS		0.015	−19.2	0.612	−2.1			
S11	2.17_469.1666m/z	HILIC -	2.17	469.1666	59, 253, 281, 369, 469		0.019	−2.5	0.763	−0.7			
S12	1.13_629.2279m/z	HILIC -	1.13	629.2279	192, 255, 352, 381, 425, 567		0.020	−1.7	0.085	−0.8			
S14	0.72_650.2950n	HILIC -	0.72	695.3120	59, 187, 367, 653		0.023	−3.3	0.482	−1.0			
	0.63_251.1323m/z	HILIC -	0.63	251.1323	97	C11H22O4S	0.044	1.8	0.261	−0.8			
S15	0.60_326.2033n	HILIC -	0.60	307.1949	97	C14H30N2O3S	0.048	1.6	0.497	−0.8			
	0.61_346.3067n	HILIC +	0.61	364.3398	no MS/MS		0.024	1.9	0.474	−1.2			
	0.61_374.2716n	HILIC -	0.61	395.2462	97	C15H34N6O4S	0.026	1.6	0.946	−0.9			
S16	1.13_663.2641m/z	HILIC +	1.13	663.2641	103, 299, 329, 439, 495, 607, 663		0.033	−2.5	0.845	1.0			
	0.92_663.2646m/z	HILIC +	0.92	663.2645	103, 299, 329, 439, 495, 607, 663		0.031	−2.6	0.159	−1.1			
S17	13.68_616.1753m/z	RP +	13.68	616.1753	557.00000		0.033	−1.8	0.564	1.4			

S18	0.95_287.1985m/z	HILIC +	0.95	287.1985	no MS/MS		0.040	−2.5	0.266	-1.2			
S19	3.88_917.5394m/z	HILIC -	3.88	917.5394	no MS/MS		0.043	−3.7	0.856	−0.9			
S20	3.60_482.2928m/z	HILIC -	3.60	482.2927	97, 224, 422, 482		0.047	0.7	0.008	−1.8			
S21	4.14_763.5052n	HILIC +	4.14	786.4944	645, 786		0.047	−2.3	0.652	−1.0			
S22	3.90_197.0321m/z	HILIC -	3.90	197.0321	67, 122, 139, 154	C6H4N4O4	0.048	−1.9	0.696	−1.1			
S23	0.93_525.3810m/z	HILIC -	0.93	525.3810	no MS/MS		0.050	−1.7	0.507	−0.9			
S24	0.75_186.4496m/z	HILIC -	0.75	186.4496	no MS/MS		0.067	0.5	0.012	−2.3			
	0.75_187.0078m/z	HILIC -	0.75	187.0078	80, 107, 187	C7H6O4S	0.317	0.7	0.009	−1.9	Benzylsulfate	2	M-H
S25	0.77_626.5093m/z	HILIC +	0.77	626.5093	no MS/MS		0.090	1.6	0.034	1.6			
S26	4.14_477.1033m/z	HILIC -	4.14	477.1033	59, 71, 89, 113		0.145	0.8	0.037	−2.6			
S27	0.74_190.0293n	HILIC +	0.74	223.0627	73, 119, 149, 165, 191, 207		0.262	1.9	0.050	2.1			
	0.72_554.1736m/z	HILIC +	0.72	554.1735	123, 223,267, 281, 355, 554		0.586	1.0	0.009	1.7			
S28	2.48_130.0887m/z	RP -	2.48	130.0886	62, 130	C6H11NO2	0.263	1.2	0.020	2.1	Leucine	1	M-H
S29	2.40_140.1503m/z	RP -	2.40	140.1503	no MS/MS		0.310	1.1	0.004	1.6			
S30	2.01_167.0216m/z	RP -	2.01	167.0215	69, 96, 124, 167	C5H2N4O3	0.318	1.0	0.013	1.6	Uric acid	1	M-H
S31	2.42_180.0681m/z	RP -	2.42	180.0680	119	C9H9NO3	0.450	1.4	0.013	4.4	Tyrosin	1	M-H
S32	3.53_681.2453m/z	HILIC +	3.53	681.2452	299, 429, 482, 649		0.808	−5.2	0.040	−14			

Table S3. Additional features identified in serum after mixed-effect model statistics between placebo and GHB intake, sorted by different compound classes. Identification confidence was assigned based on the Metabolomics Standard Initiative (MSI) as follows: confirmation using MS/MS information and co-elution with authentic standards (level 1); confirmation through comparison of experimental MS/MS spectra with online databases (level 2); and annotation to putatively characterized compound classes (level 3). RT, retention time; *m/z* mass to charge ratio; *p* 4.5/16.5 *p*-value at time-point 4.5 h and 16.5 h, respectively; MM, mixed effect model; FC foldchange; HILIC hydrophilic interaction liquid chromatography; RP reversed phase.

p MM 4.5	*p* MM 16.5	Compound	Method	RT	*m/z*	*p* 4.5	FC 4.5	*p* 16.5	FC 16.5	(Tentative) Identification	.	Adduct
0.000	0.013	0.85_104.1062m/z	RP +		104.1061502	0.001	0.9	0.055	0.9	Choline	1	M+H
0.027	0.017	0.94_496.2565n	HILIC -		541.2594178	0.050	1.1	0.066	1.1	Leukotriene D4	2	M-H, M+FA-H
0.013		0.80_173.1053m/z	RP -		173.1053479	0.122	0.9	0.539	1.0	Arginine	1	M-H
0.011		0.78_133.0383n	RP -		154.0627341	0.014	0.9	0.970	1.0	Aspartic acid	1	M-H, M+Na-2H
0.004		0.77_156.0767m/z	RP +		156.0767447	0.016	0.9	0.632	0.9	Histidine	1	M+H
0.029		4.41_164.0719m/z	RP -		164.0718582	0.082	1.0	0.998	1.0	Phenylalanine	1	M-H
0.014		5.54_203.0831m/z	HILIC -		203.0831	0.015	0.8	0.463	1.0	Tryptophan	1	M-H
0.001		9.86_129.0790n	HILIC +		147.1121256	0.004	0.9	0.166	0.9	Pipecolic acid	2	M+H, M+NH4
0.021		0.90_162.1125m/z	RP +		162.1125384	0.003	0.9	0.030	0.9	Carnitine	1	M+H
0.035		15.30_426.3549m/z	RP +		426.3548814	0.158	1.0	0.606	0.9	Oleoylcarnitine	2	M+H
	0.049	0.78_314.2443n	HILIC +		279.2304467	0.964	1.0	0.026	1.1	9,10-DHOME	2	M+H-2H2O, M+H-H2O, M+H
0.032		16.30_307.2634m/z	RP -		307.2633971	0.348	1.4	0.861	0.9	Dihomo-linoleate (20:2n6)	2	M-H
0.047		0.96_280.2398n	HILIC +		313.2712206	0.120	1.1	0.864	1.1	Linoleic acid	1	M+H-2H2O, M+CH3OH+H
0.033		13.71_272.2572m/z	RP +		272.2571851	0.051	1.0	0.695	1.0	Palmitoleic acid	2	M+NH4
0.033		1.97_407.2794m/z	HILIC -		407.2794052	0.439	0.7	0.475	0.7	Cholic acid	1	M-H
0.001		3.60_563.2593n	HILIC -		562.2510334	0.001	0.8	0.098	0.9	Taurolithocholic acid 3-sulfate	2	M-H, M+K-2H, M-2H
0.019		5.21_480.3092m/z	HILIC -		480.3092411	0.033	0.9	0.462	1.0	LysoPC(15:0)	2	M-H
	0.030	15.18_494.3249m/z	RP +		494.3249002	0.545	0.9	0.257	1.0	LysoPC(16:1(9Z))	2	M+H
0.003		5.16_508.3398m/z	HILIC -		508.3398452	0.013	0.9	0.515	1.0	LysoPC(17:0)	2	M-H
0.021		5.16_543.3384n	HILIC -		578.3077843	0.069	0.8	0.400	1.2	LysoPC(20:4(8Z,11Z,14Z,17Z))	2	M+Cl, M+K-2H
0.012		5.08_507.3657n	HILIC -		542.3350839	0.020	0.8	0.170	1.3	LysoPC(P-18:0)	2	M+Cl, M+FA-H
0.034		5.23_516.2879m/z	HILIC -		516.2878698	0.031	0.9	0.455	0.9	LysoPE(18:0/0:0)	2	M+Cl
0.039		15.36_478.2934m/z	RP +		478.2933651	0.092	1.2	0.533	1.0	LysoPE(18:2(9Z,12Z)/0:0)	2	M+H
0.003		15.53_502.2927m/z	RP -		502.2926645	0.016	1.1	0.363	1.0	LysoPE(20:3(8Z,11Z,14Z)/0:0)	2	M-H

0.019	4.66_524.2775m/z	HILIC -	524.2775147	0.020	1.1	0.791	1.0	LysoPE(22:6(4Z,7Z,10Z,13Z,16Z,19Z)/0:0)	2	M-H
0.034	4.67_500.2777m/z	HILIC -	500.2776585	0.044	1.2	0.898	1.0	PE(20:4/0:0)	2	M-H
0.002	4.66_526.2917m/z	HILIC -	526.2916714	0.027	1.1	0.758	1.0	PE(22:5/0:0)	2	M-H

Materials and methods

Chemicals and Reagents

1-Methylhistidine, adenine, adenosine, arginine, azelaic acid, butyrylcarnitine, carnitine, chenodeoxycholic acid, cholic acid, citrulline, cortisol, cortisone, creatinine, deoxycholic acid, glutaric acid, glycolic acid, glycocholic acid, hippuric acid, inosine, isoleucine, leucine, L-pyroglutamic acid, methionine, methylmalonic acid, mevalonolactone, N,N-dimethylglycine, nicotinic acid, p-aminobenzoic acid, phenylalanine, proline, raffinose, riboflavin, taurine, taurocholic acid, tryptophan, and uracil were purchased from Sigma-Aldrich (Buchs, Switzerland). Deuterated and heavy-labeled internal standards (IS) adenosine ribose-D_1, arginine-$^{13}C_6$, caffeine 3-methyl-^{13}C, carnitine trimethyl-D_9, creatinine N-methyl-D_3, deoxycholic acid-D_4, D-fructose ^{13}C, glycine-$^{13}C_2$, glycocholic acid-D_4, hippuric acid ^{15}N, kynurenine-D_4, leucine-D_{10}, lysine-D_4, phenylalanine-D_1, proline ^{15}N, serine-D_3, tryptophan-D_5 and uric acid-$^{15}N_2$ were purchased from Cambridge isotope laboratories, which were delivered by ReseaChem Life Science (Burgdorf, Switzerland) or Sigma-Aldrich (Buchs, Switzerland). GHB-carnitine was synthesized and delivered by Toronto Research Chemicals (Toronto, Canada). Water, acetonitrile (ACN), methanol (MeOH) of HPLC grade were obtained from Fluka (Buchs, Switzerland). All other chemicals used were from Merck (Zug, Switzerland) and of the highest grade available.

UHPLC-HRMS

MS measurements were performed in randomized order in three batches (serum, urine Ut1, urine Ut2) on a Thermo Fischer Ultimate 3000 UHPLC system (Thermo Fischer Scientific, San Jose, CA) coupled to a HR TOF instrument system (TripleTOF 6600, Sciex, Concord, Ontario, Canada) Two different columns—RP (Waters XSelect HSST RP-C18 column (150 mm × 2.1 mm, 2.5 µm particle size)) and HILIC (Merck SeQuant ZIC HILIC column (150 mm × 2.1 mm, 3.5 µm particle size)) were used for chromatographic separation using gradient elution with mobile phases A and B (10 mM ammonium formate with 0.1% (*v*/*v*) formic acid in water and 0.1% (*v*/*v*) formic acid in MeOH) and C and D (25 mM ammonium acetate and 0.1% (*v*/*v*) acetic acid in water and 0.1% (*v*/*v*) acetic acid in ACN), respectively. The column oven was set to 40 °C and injection volume was 1 µL for all samples.

HR MS and MS/MS data were acquired by two methods: TOF MS only and information dependent data acquisition (IDA) in positive and negative ionization mode. MS analysis was performed with a DuoSpray ion source at a resolving power (full width at half-maximum at *m*/*z* 400) of 30,000 in MS1 and 30,000 in MS2 (high-resolution mode) or 15,00 (high-sensitivity mode) in positive ionization mode. Automatic calibration was obtained every fifth sample injections using atmospheric-pressure chemical ionization (APCI) positive calibration solution (Sciex) in the positive ionization mode and every three sample injections using APCI negative calibration solution (Sciex) in the negative ionization mode. The TOF MS method was composed of a TOF-MS scan over a mass range from *m*/*z* 50 to *m*/*z* 1000 (accumulation time 100 ms, collision energy (CE) 5 eV). Additionally, about 20% of the samples were measured in the IDA scan mode. The IDA method consisted of a TOF-MS scan over a mass range from m/z 50 to *m*/*z* 1000 (accumulation time 50 ms, CE 5 eV). IDA experiments (accumulation time for each IDA experiment 100 ms, CE 35 eV with a CE spread of 15 eV) were performed after dynamic background subtraction on the four most intense ions with an intensity threshold above 100 counts per second (cps) and exclusion time of 5 s (half peak width) after two occurrences in high sensitivity mode.
